# Population Genetic Analyses of the Fungal Pathogen *Colletotrichum fructicola* on Tea-Oil Trees in China

**DOI:** 10.1371/journal.pone.0156841

**Published:** 2016-06-14

**Authors:** He Li, Guo-Ying Zhou, Jun-Ang Liu, Jianping Xu

**Affiliations:** 1 The Ministry of Education Key Laboratory for Non-Wood Forest Cultivation and Conservation, Central South University of Forestry and Technology, Changsha, China; 2 College of Forestry, Central South University of Forestry and Technology, Changsha, China; 3 Department of Biology, McMaster University, Hamilton, Ontario, Canada; Università Politecnica delle Marche, ITALY

## Abstract

The filamentous fungus *Colletotrichum fructicola* is found in all five continents and is capable of causing severe diseases in a number of economically important plants such as avocado, fig, cocoa, pear, and tea-oil trees. However, almost nothing is known about its patterns of genetic variation and epidemiology on any of its host plant species. Here we analyzed 167 isolates of *C*. *fructicola* obtained from the leaves of tea-oil tree *Camellia oleifera* at 15 plantations in seven Chinese provinces. Multilocus sequence typing was conducted for all isolates based on DNA sequences at fragments of four genes: the internal transcribed spacers of the nuclear ribosomal RNA gene cluster (539 bp), calmodulin (633 bp), glutamine synthetase (711 bp), and glyceraldehyde-3-phosphate dehydrogenase (190 bp), yielding 3.52%, 0.63%, 8.44%, and 7.89% of single nucleotide polymorphic sites and resulting in 15, 5, 12 and 11 alleles respectively at the four gene fragments in the total sample. The combined allelic information from all four loci identified 53 multilocus genotypes with the most frequent represented by 21 isolates distributed in eight tea-oil plantations in three provinces, consistent with long-distance clonal dispersal. However, despite evidence for clonal dispersal, statistically significant genetic differentiation among geographic populations was detected. In addition, while no evidence of recombination was found within any of the four gene fragments, signatures of recombination were found among the four gene fragments in most geographic populations, consistent with sexual mating of this species in nature. Our study provides the first insights into the population genetics and epidemiology of the important plant fungal pathogen *C*. *fructicola*.

## Introduction

Domestication of natural ecosystems and the expanding trade of goods and services have accelerated the emergence and spread of pathogens [1,2]. In agriculture and forestry, widely distributed and genetically homogenous crop genotypes are conducive for rapid emergence and spread of plant pathogens across large areas. Investigations into the patterns of genetic variation and their potential routes of dispersal are crucial for developing strategies for the control and prevention of infectious disease epidemics. This is especially true for pathogens capable of long-distance dispersal, for which the emergence of advantageous mutations such as increased virulence and/or resistance to fungicides could spread rapidly over large geographical areas [3].

The ascomycete fungal genus *Colletotrichum* contains over 60 recognized species and likely many more cryptic (still to be described) species [4–7]. The genus is distributed throughout the tropical, subtropical and temperate regions of the globe and causes some of the most devastating diseases in plants including major groups of agricultural crops such as cereals, vegetables and fruits, especially in tropical and subtropical regions [4,7,8]. Species in this genus can cause diseases in both pre- and post- harvest stages [7,9]. In the presence of appropriate nutrients, moisture and ambient temperature, these organisms can produce abundant asexual spores that could disperse long distances. Among the species in this genus, 22 species constitute the *Colletotrichum gloeosporioides* species complex (CGSC) [7]. This species complex can infect a diversity of plant organs, including stems, leaves, flowers and fruits and cause diseases in many vegetables and fruits, including peppers, cocoa, oranges, apples, bananas, mangos, ramie, mulberry, pistachio, persimmon, strawberries, and tea-oil trees [7–19]. The most common disease is anthracnose that can result in 30–50% of crop failure [9,12].

*Colletotrichum fructicola* is a recently described species within CGSC [11]. Morphologically, *C*. *fructicola* is very similar to or indistinguishable from others within CGSC and molecular markers are needed in order to separate it from other closely related species [7,11]. Despite its short history of recognition by fungal taxonomists and plant pathologists, *C*. *fructicola* has been found to cause diseases in many plants, including fruit rot in *Persea americana* and *Coffea arabica* as well as leaf anthracnose in *Malus domestica*, *Fragaria × ananassa*, *Ficus edulis*, *Limonium sinuatum*, *Pyrus pyrifolia*, *Dioscorea alata*, *Dioscorea rotundata*, *Tetragastris panamensis*, and *Camellia sinensis* [7,11]. In addition, *C*. *fructicola* is geographically broadly distributed—it has been reported on all five continents, throughout the Americas, Western and Eastern Asia, Western Europe, Western Africa, and Australia [7,11]. However, despite its ecological and economic importance, very little is known about its epidemiology and population biology.

One of the major host plants of CGSC in China is the tea-oil tree *Camellia oleifera* [10,12]. *C*. *oleifera* is native to China and is cultivated in many parts of southern China [12,20,21]. This plant has been grown for over two thousand years, mainly for its high-quality cooking oil [21]. *C*. *oleifera* has a deep taproot system and can grow in a diversity of niches, including steep hills with relatively poor nutrients [12,20–22]. As a result, there have been significant efforts in recent years to expand the cultivation of *C*. *oleifera* for both food (i.e. oil) production and environmental protection (e.g. preventing soil erosion) [22]. At present, most such plantations in China are situated on sloppy hills of poor-quality soil not suitable for most other agricultural crops [12,22]. These plantations cover approximately 30,000 square kilometers and produce 250,000 tons of edible oil each year [12,22]. The high-quality cooking oil is extracted from the seeds of mature fruits. Chemical analyses of the tea-oil identified that it contained over 80% monounsaturated fat and as such [22], it could help reduce LDL (the “bad cholesterol”) in humans. Aside from its edibility and health benefits, tea-oil has also been used in textile manufacturing, soap making, and woodwork protection [22].

The expanding cultivation of *C*. *oleifera* over the last few decades has also attracted increasing attention of plant pathologists to infectious diseases on this plant. While historical records are not available, recent estimates suggested that up to 40% of *C*. *oleifera* yield were lost in some plantations due to CGSC anthracnose [10,12]. Strains of CGSC can infect the fruits, buds, and leaves of *C*. *oleifera* plants, resulting in premature leaf and fruit rot and drop as well as wilting of the leaves and buds. In southern China where most of this species is artificially cultivated, anthracnose can occur from early April to late October, peaking in August [10,12]. However, despite the severity of anthracnose on *C*. *oleifera*, relative little is known about its molecular epidemiology, including the causative agent(s), its potential dispersal pattern, the relationships among geographic populations, and its modes of reproduction in nature.

**Fig 1:**
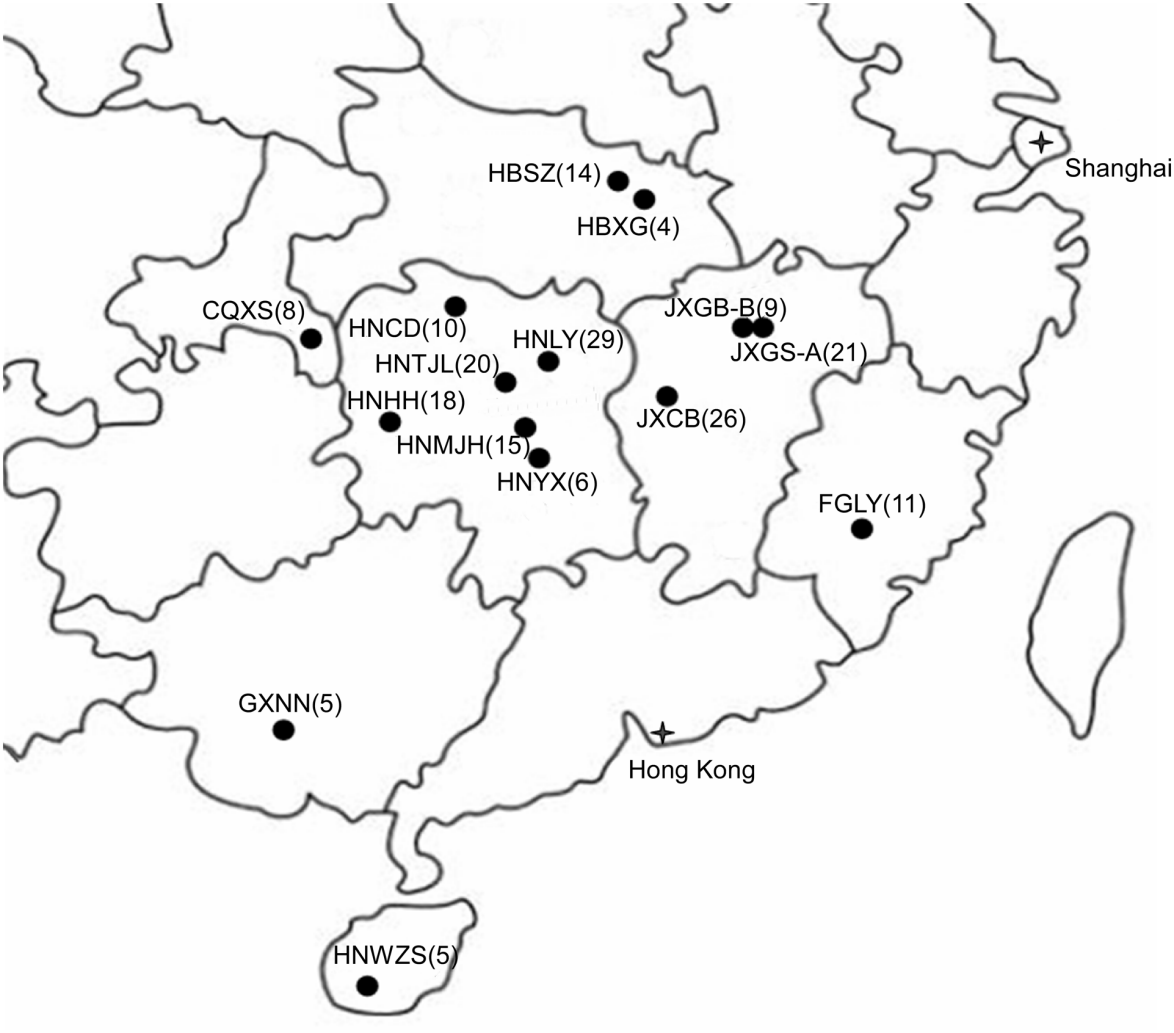
An outline map showing sampled tea-oil tree plantations. This map shows the geographic locations in southern China where samples of the *Colletotrichum gloeosporioides* species complex from tea-oil tree plantations were obtained and analyzed in this study. Number of isolates obtained from each plantation was indicated in parenthesis.

Since the mid-1990s, multilocus sequence typing (MLST) [23] and multiple gene genealogical analyses (MGGA) have been increasingly used in microbial evolutionary studies [24–27]. Indeed, such analyses have not only helped identify cryptic species in many fungal taxa and revealed the evolutionary relationships among distinct taxa [7,24] but also facilitated our understanding of the population genetics and molecular epidemiology of many animal and plant fungal pathogens [25,26,28–30]. In the case of the *C*. *gloeosporioides* species complex, MGGA has helped define the 22 phylogenetic species within this species complex [7]. Here we used MLST and MGGA at four loci (*ITS*, *CL*, *GS*, and *GD*) to analyze strains of CGSC from anthracnose-infected leaves of tea-oil trees from 15 plantations in 7 southern Chinese provinces (Fig 1).

Among the 199 isolates of CGSC that we obtained, 167 were found belonging to *C*. *fructicola*. The genotype data at the four loci for the 167 isolates were analyzed to address the following specific issues, (i) evidence for clonality and recombination within individual populations (A sexual morph *Glomerella cingulata* var. *minor* has been found for *C*. *fructicola* [31,32], thus we hypothesize that signatures of recombination will be found in natural populations of *C*. *fructicola*.), (ii) the extent to which clones are spread among the geographic populations of *C*. *fructicola* in southern China, and (iii) the genetic relationships among the geographic populations, including potential evidence of genetic isolation by geographic distance.

## Materials and Methods

### Fungal Isolates

A total of 750 *C*. *oleifera* infected leaves with symptoms of anthracnose were obtained, including 50 leaves from each of the 15 tea-oil tree plantations in seven provinces in southern China (Fig 1). Within each plantation, each of the 50 leaves was from a different tea-oil tree in an area of ~100 m X ~100 m. The sampled trees/leaves per province ranged from 50 (Chongqing, Fujian, Guangxi, Hainan, one plantation each), to 100 (Hubei, two plantations), 150 (Jiangxi, three plantations), and 300 (Hunan, six plantations) (Table 1). The field studies did not involve endangered or protected species and no specific permissions were required to collect diseased tea-oil tree leaves from these locations. The geographic coordinates for all 15 plantations are provided in Table 1. These tea-oil tree plantations differed in their time of establishment and age (Table 1). Two of the sites (Changbu in Jiangxi and Tianjiling in Hunan) are *C*. *oleifera* germplasm centers where hundreds of cultivar lines have been maintained at each plantation since the 1960s. Each of the remaining 13 plantations consisted of one or a few tea-oil tree cultivars at each plantation.

**Table 1:** Summary information of *C*. *fructicola* samples infecting leaves of the *Camellia oleifera* trees in southern China.

Tea-oil tree plantations: Site, Province (site abbreviations)	Latitude (° North)	Longitude (° East)	Age of plantation (years)	No. of CGSC isolates	No. of *C*. *fructicola* isolates	*C*. *fructicola* allelic diversity^1^ (Mean and range of allele number per locus)	Total no. of private alleles^2^	No. of *C*. *fructicola* multilocus genotypes
Guangshang, Jiangxi (JXGS-B)	28.014	115.657	~35	9	6	0.300 (1.75, 1–2)	1	4
Guangshang, Jiangxi (JXGS-A)	28.016	115.659	~50	21	19	0.382 (3.00, 1–5)	1	13
Changbu, Jiangxi (JXCB)	27.564	114.626	~50	26	24	0.381 (3.25, 1–7)	2	12
Wuzhishang, Hainan (HNWZS)	18.78	109.52	4	5	5	0.300 (1.75, 1–3)	3	4
Youxian, Hunan (HNYX)	27.01	113.32	~40	6	6	0.400 (2.00, 1–3)	1	5
Tianjiling, Hunan (HNTJL)	28.113	113.049	~50	20	12	0.436 (3.50, 1–5)	7	8
Majiahe, Hunan (HNMJH)	27.818	113.043	~35	15	11	0.482 (2.75, 2–4)	1	7
Liuyang, Hunan (HNLY)	28.364	113.448	~30	27	27	0.372 (3.50, 1–7)	3	12
Huaihua, Hunan (HNHH)	28.533	110.967	~30	18	15	0.412 (3.00, 1–6)	1	11
Changde, Hunan (HNCD)	29.02	111.51	~100	10	4	N/C (2.50, 2–3)	2	4
Xiaogang, Hubei (HBXG)	31.417	114.617	~30	4	4	N/C (2.00, 1–3)	0	4
Suizhou, Hubei (HBSZ)	32.267	113.431	5	14	14	0.382 (2.50, 1–4)	3	9
Nanning, Guangxi (GXNN)	22.927	108.354	~30	5	2	N/C (1.00, 1–1)	0	1
Longyan, Fujian (FJLY)	25.379	117.301	~50	11	11	0.327 (2.25, 1–4)	1	6
Xiushan, Chongqing (CQXS)	28.75	109.633	~80	8	7	0.345 (2.00, 1–3)	2	4

^1^ Allelic diversity = (N/(N-1)) * (1—Sum pi^2) where pi is the frequency of the *i*th allele for the population. N/C, not calculated due to small sample sizes.

^2^ a private allele refers to an allelic sequence found only in one tea-oil tree plantation. The private alleles can be from any of the four sequenced gene fragments. The number shown in this table refers to the sum of private alleles from all four loci in each of the plantations.

Isolation of pure cultures of *C*. *fructicola* and its close relatives followed methods described previously for isolating strains of CGSC [5,8,10,32]. In brief, each harvested leaf with anthracnose symptoms was first washed with sterile distilled water. From each leaf, a 3mm x 3mm section of the infected area was cut with a sterile knife and surface-sterilized with 70% alcohol for 45 seconds. This was followed by soaking the leaves in 0.1% mercury chloride solution for 45 seconds and subsequently three washes with sterilized distilled water at 1 minute each. Each leaf tissue was then placed on the surface of a potato-dextrose-agar (PDA) medium in a Petri-dish and incubated at 25°C under constant light at a relative humidity of 80% until mycelia grew from the edge of leaf tissue onto the agar medium. This protocol was used in order to minimize microbial contaminants from the leaf surface during our isolation. Hyphal tip cultures were then transferred onto new PDA plates and incubated for 10 days at 25°C. To ensure that our analyzed cultures were pure, a monoconidial culture was then established from each culture on PDA plates and incubated for 10 days at 25°C. The isolated fungal cultures that contained the characteristic macroscopic and microscopic (mycelia and conidia) features of *C*. *fructicola* as well as its close relatives within CGSC [4,8] were stored at -80°C until further analyses.

### Multilocus Sequence Typing

The total genomic DNA was isolated from each strain using a protocol described previously [33]. The quality and quantity of the DNA were determined using a SmartSpec^™^ Plus spectrophotometer (Bio-Rad Laboratories) and agarose gel electrophoresis. The DNA samples were diluted to 0.5 μg/ml and used in PCR reactions in order to amplify: (i) the internal transcribed spacer (ITS) regions of the nuclear ribosomal RNA gene cluster; (ii) calmodulin (CL); (iii) glutamine synthetase (GS); and (iv) glyceraldehyde-3-phosphate dehydrogenase (GD). These markers have been used in previous studies and shown to be effective for discriminating strains and species within CGSC [7,8,11]. Each PCR reaction was carried out in a total volume of 16 μl [27] and the mixture was composed by 2μl (~1 ng) of the diluted genomic DNA, GoTaq Master mix (2X) (Promega), 0.2μM of each primer, whose sequences were described previously [7,11]. The thermo cycling profile included an initial denaturizing step at 95°C for 4 min to ensure that all DNA was denatured to start the reactions. This was followed by 40 cycles of: denaturation at 95°C for 1 min, primer-specific annealing temperature for 1 min, and DNA synthesis at 72°C for 1 min. This was then followed by a final extension at 72°C for 7 min. PCR products were purified using the QIAGEN DNA Purification Kit and sequenced using both the forward and reverse primers at Shanghai Sangong Biotech Company, using the ABI3100 automated DNA sequencer (Applied BioSystems, Inc./Life Technologies). The consensus nucleotide sequences were used to determine their species affiliations and to study the patterns of population genetic variation.

### Species Identification

To determine the species identity of the 199 isolates that we obtained in this study, we first used BLASTN analyses of the sequences at each individual locus against GenBank database to investigate whether the isolates belonged to the *Colletotrichum gloeosporioides* species complex (CGSC). However, as shown in previous studies [7,8,11], sequences at individual locus are insufficient to discriminate closely related species within CGSC. Instead, we followed the phylogenetic approaches as recommended by previous studies [7,11] and used the concatenated sequences of three gene fragments to resolve the species status of our strains. Specifically, we compared the sequences of our isolates to those representing the 22 individual phylogenetic species within CGSC. Because only three of the four DNA fragments (the *ITS*, *CL* and *GD*) were found in all the type or epitype strains representing the 22 known species within CGSC [7,11], our species identification used these three gene fragments. Briefly, orthologous sequences at all three loci from our samples and those of authenticated strains representing individual phylogenetic species within CGSC [7,11] were retrieved from GenBank. Sequences at individual loci were aligned using Clustal X version 2 [34,35]. The alignments were manually inspected and adjusted if necessary. The concatenated sequences of the three gene fragments were analyzed using the Neighbor-Joining algorithm implemented in the MEGA software [36,37], following procedures used in previous studies of this species complex [7,11]. The GenBank accession numbers for our sequences are KJ131572-KJ131770 for *ITS*; KJ131771-KJ131969 for *GD*; KJ131970-KJ132168 for *GS*; and KJ132169-KJ132367 for *CAL* gene fragments. S1 Table presents the detailed GenBank accession numbers for each of the 796 sequences generated in this study, including the detailed isolate names, their geographic origins, and species affiliations.

### Nucleotide Sequence Polymorphism and Genetic Diversity

Among the 199 CGSC isolates, 167 belonged to *C*. *fructicola* while the remaining 32 isolates belonged to 3–4 other species (See Results below). The within- and between-species mean pairwise strain genetic distances were calculated using the MEGA software [36,37]. Similarly, the within- and between- species nucleotide diversities were obtained through MEGA. However, due to the small sample sizes of the non—*C*. *fructicola* species in our total sample, their population genetic analyses were not performed. Instead, we focused on geographic populations of *C*. *fructicola*. The computer program GenAlEx 6.5 [38] was used to obtain the population genetic parameters such as the number of polymorphic sites within each gene fragment, and the number of alleles and allelic diversity at each gene fragment within individual geographic populations. For the three protein-coding genes (*GD*, *GS*, and *CAL*), their dN/dS ratios were calculated using the MEGA software [36,37].

### Mode of Reproduction

In natural environments, all microbes can reproduce asexually through budding, fission, and for filamentous fungi, hyphal extension and/or asexual sporulation. Baring mutation, asexually reproduced progeny would have an identical genotype as the parental cell. The clonally derived progeny cells could disperse to different ecological niches and geographic locations. However, mutations could accumulate during asexual reproduction and form clonal lineages. Indeed, evidence for such clones and clonal lineages has been found in all microbial species examined so far, including many fungal pathogens of animals and plants [25,26, 39, 40]. In CGSC, the dispersal of asexually reproduced spores and hyphae would result in strains with identical or very similar genotypes from different tea-oil trees, plantations and geographic areas. While clonality is known to be a significant component of microbial populations in nature [23,25], the role of recombination in most microbial populations is often unknown.

Recombination shuffles alleles and generates novel genotypes. At the population level, a signature of recombination is the lack of strong association among alleles at different loci. Thus, to identify whether there was recombination in natural microbial populations, two complementary statistical measures have often been used: phylogenetic incompatibility [41] and the multilocus linkage equilibrium called the Index of Association (*I*_*A*_ or a modified measure of *I*_*A*_ called *rd* which is a standardized *I*_*A*_ by the number of loci) [42]. These two tests have different null hypotheses. The null model for *I*_*A*_ is random recombination while that for phylogenetic incompatibility is strict asexual reproduction and clonality. The underlying principles, methods of calculations and interpretations of the results for both tests are described in the Multilocus program manual [41]. Here we used these two tests implemented in the Multilocus program to identify potential signatures of recombination.

In natural populations, recombination may occur among nucleotide sites within individual DNA fragments [27,43]. If so, individual nucleotide sites should be treated as independent loci to examine the extent of recombination between these DNA fragments. In contrast, if there is no evidence of recombination among nucleotide sites within individual DNA fragments, each sequenced fragment should be treated as an individual locus in the analyses of recombination between the DNA fragments. Otherwise, the within-locus linkage disequilibrium among variable nucleotide sites would contribute to over-estimates of clonality in natural microbial populations [27,43]. To determine which data type should be used to examine evidence of recombination in natural populations of *C*. *fructicola*, we first examined whether there is evidence for recombination within each of the four sequenced gene fragments using the total sample of 167 isolates. Both the index of association and phylogenetic incompatibility tests were conducted.

Our tests revealed no evidence of recombination among variable nucleotide sites within any of the four sequenced DNA fragments (see Results below). Thus, our test of recombination among loci will use allelic information based on the entire DNA sequences at each of the four fragments. Specifically, our tests for evidence of recombination were conducted for three sample types of *C*. *fructicola*: (i) individual geographic populations; (ii) the total sample that included all 167 isolates; and (iii) the clone-corrected total sample of 53 unique genotypes. The third sample type was analyzed to examine, after excluding an obvious clonal component (i.e. isolates sharing the same genotype), whether the signatures of recombination would be more evident. For the analyses of individual geographic populations, only the eight populations (Table 1) with sample sizes greater than 10 in each were tested.

### Relationships among Geographic Populations

We analyzed the genetic relationships among populations of *C*. *fructicola* from the tea-oil tree plantations. The program GenAlEx 6.4 [38] was used to determine the extent of genetic differentiation between the geographic populations. However, three of the 15 geographic populations (Nanning in Gunagxi, Changde in Hunan, and Xiaogang in Hubei) had small sample sizes of *C*. *fructicola* (less than five in each, Table 1). As a result, our population genetic analyses using the GenAlEx program excluded these three samples and focused on the remaining 12 geographic populations. Both pairwise population differentiation (F_ST_) and total genetic differentiation based on the analysis of molecular variance (AMOVA) were calculated [44,45]. Principal component analyses (PCA) of the pairwise F_ST_ values were used to identify major axes separating the geographic populations. The statistical significance of the *F*_*ST*_ and AMOVA results was obtained by comparing the distribution of the observed values to the expected distribution based on re-sampled data (1000 times in our study) while assuming no differentiation. During re-sampling to generate the expected distribution, the subpopulation sizes were held constant but individuals were reassigned to different subpopulations to simulate gene flow to generate genetically undifferentiated subpopulations. Furthermore, the relationships between geographic distance and genetic distance among geographic populations were examined using the Mantel test implemented in the GenAlEx 6.4 program [38]. If obvious outliers with small sample sizes were identified in the initial analyses, these outliers were excluded and the remaining samples were further analyzed, following procedures described above.

In addition to the above pre-defined, geography-based analyses, we also used STRUCTURE 2.3.4 to identify the number of genetically distinct clusters (K) in our sample and to assign individual isolates to specific clusters (K) [45,46]. STRUCTURE implements a clustering algorithm based on a Bayesian Monte Carlo Markov Chain (MCMC) approach to assign individuals into K distinct populations. Here, all SNPs were included in the analysis. Using the admixture model, 10 replicated runs each of K = 1–15 were carried out, with each of the 150 runs having a burn-in period of 100,000 generations followed by a run length of 1,000,000 generations. K = 15 was set as the upper limit based the number of tea-oil tree plantations that we sampled. These run parameters used here followed those recommended in a recent meta-study that analyzed the robustness and reproducibility of STRUCTURE output results [47]. Our outputs of 150 runs from STRUCTURE were processed using CLUMPAK developed by Evanno et al. [48] to generate the optimal number of genetically distinct clusters (K) in our sample.

As described above, two (HNTJL and JXCB) of the 15 sampled plantations were tea-oil tree breeding centers. We were interested in whether these two plantations also had higher genetic diversities of the fungal pathogen *C*. *fructicola* than other plantations and whether they could potentially be the centers of *C*. *fructicola* diversification. To examine the possibilities, we employed two tests. In the first, we compared the number of alleles, the number of private alleles, and the allelic diversities using the Wilcoxon signed-rank test (a non-parametric paired rank order test) between these two plantations and the remaining 10 plantations each with a sample size greater than 5 (i.e. excluding the GXNN, HNCD, and HBXG samples). In the second test, we used the DIYABC program [49] to compare two simple alternative scenarios about the evolutionary history of *C*. *fructicola* populations in tea-oil tree plantations. In the first scenario, the HNTJL and JXCB populations of *C*. *fructicola* served as ancestral populations and were the first two to diverge and the other 10 geographic populations subsequently diverged from them. In the second scenario, all samples of *C*. *fructicola* from the 12 plantations diverged at the same time. Default parameters (e.g. equal mutation rates among the four genes and equal sex ratios within each population) were used to generate the simulated data for posterior probability calculations of the two scenarios. 1000 simulations were done to help derive statistical significance of the difference between the two scenarios.

## Results

A total of 199 isolates of *C*. *fructicola* and its close relatives within the C. *gloeosporioides* species complex were isolated from the 750 leaf samples (each leaf was from a different tea-oil tree), representing a 26.5% overall success rate. However, the success rates varied widely among the geographic samples (Table 1), from less than 10% in Xiaogang, Hubei to over 50% in Liuyang, Hunan. We successfully obtained the DNA sequences from all 199 isolates at the four gene fragments. The GenBank accession numbers for the 796 sequences generated in this study are presented in S1 Table.

At these four loci, no evidence of heterozygosity was found for any of the 199 isolates, consistent with their haploid status found in previous studies [7,11]. Phylogenetic analysis of the aligned sequences identified the relationships among the strains for each of the four individual gene fragments (S2 Table and S1–S4 Figs). Due to the large sample size, the strain relationships are not shown for all 199 isolates at each of the four loci in S1–S4 Figs. Instead, to allow easy visualizations, only distinct alleles from each geographic population were included to show the relationships among the distinct alleles at each of the four loci (S1–S4 Figs). However, the number of isolates that shared the same allele from each plantation was indicated and shown in the four supplementary figures. The GenBank accession numbers and the allelic designations for each isolate are presented in S1 and S2 Tables.

### Phylogenetic Species Identification

**Fig 2:**
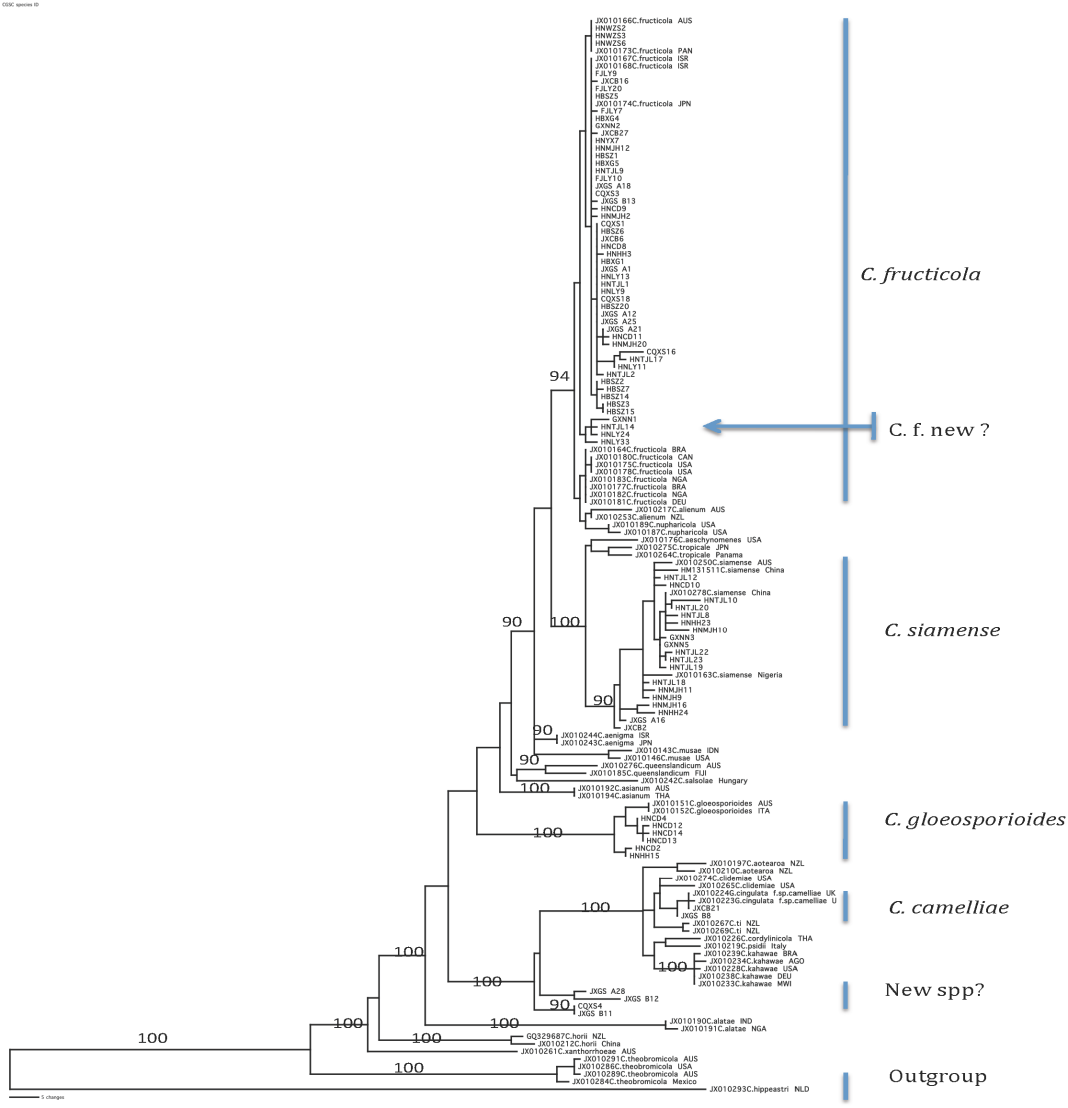
Neighbor-Joining phylogeny showing the relationships among the representative strains of the 85 multilocus genotypes of the *Colletotrichum gloeosporioides* species complex (CGSC) analyzed in this study and with reference genotypes of 22 phylogenetic species within CGSC. The phylogeny was obtained by using the concatenated sequences of three loci (*ITS*, *CL and GD*) totaling 1362bp. Each reference taxon is represented by the GenBank accession number of its ITS sequence, followed by the species name and the country where the strain was originally isolated. The branch lengths are proportional to the amount of nucleotide sequence divergence. The species that our isolates belonged to were indicated at the right margin: C.f., *C*. *fructicola*; C.s., *C*. *siamense*; C.g. *C*. *gloeosporioides; C*.*c*., *C*. *camelliae*. The four genotypes representing five strains that are not clustered with any of the 22 known phylogenetic species within CGSC are marked by “New spp?”. Similarly, the seven isolates (four genotypes) within *C*. *fructicola* that are distinctly different from the remaining 160 *C*. *fructicola* isolates are marked by “C.f. new?”. Bootstrap values greater than 90% are shown. The tree was rooted by outgroup taxon *C*. *hippeastri*.

Following the procedure recommended by Prihastuti et al. [11] and Weir et al. [7], our phylogenetic analyses identified that the 199 isolates were grouped into four known phylogenetic species: *C*. *fructicola* (167 isolates), *C*. *siamense* (19 isolates), *C*. *gloeosporioides* (6 isolates), and *C*. *camelliae* (2 isolates). However, five isolates (GXNN1, CQXS4, JXGSA28, JXGSB11, JXGSB12) did not have clear phylogenetic affiliations with any of the 22 known species (Fig 2). The geographic distributions of these species and the five unassigned isolates are summarized in Table 2. The mean genetic distances (i.e. 1—nucleotide identity) between strains from within and among the three main species in our samples (*C*. *fructicola*, *C*. *siamense*, and C. *gloeosporioides*) are shown in Table 3. Our results indicated that the mean genetic distances among the three species were significantly greater than those within each of the species (P<0.001). The within- and between-species nucleotide diversities showed a similar pattern, with significantly greater nucleotide diversities between the species than within individual species (Table 3). The high standard deviations in genetic distances between strains from within and between pairs of species suggest significant genetic variations within each individual species, consistent with our observations of the high allelic and high genotypic diversities within each of the three species.

**Table 2:** Geographic distributions of phylogenetic species of CGSC from tea-oil tree leaves in southern China. The geographic abbreviations correspond to those in Table 1.

Plantation	*C*. *fructicola*	*C*. *siamense*	*C*. *gloeosporioides*	*C*. *camelliae*	*Unclassified*
GXNN	2	2			1
HNWZS	5				
FJLY	11				
CQXS	7				1
HBSZ	14				
HBXG	4				
JXGS-A	19	1			1
JXGS-B	6			1	2
JXCB	24	1		1	
HNTJL	12	8			
HNMJH	11	4			
HNYX	6				
HNCD	4	1	5		
HNLY	27				
HNHH	15	2	1		
Total	167	19	6	2	5

**Table 3:** Nucleotide diversities (π) and pairwise strain genetic distances within and among the three most common species of the *Colletotrichum gloeosporioides* species complex causing leaf anthracnose on tea-oil trees in southern China.

Species comparisons	Genetic distance^1^	Nucleotide diversity (π)^2^
Within *C*. *fructicola*	0.249 ± 0.190 (N = 2080)	0.00238
Within *C*. *siamense*	0.474 ± 0.211 (N = 253)	0.00774
Within *C*. *gloeosporioides*	0.337 ± 0.167 (N = 28)	0.00457
Between *C*. *fructicola* and *C*. *siamense*	1.955 ± 0.713 (N = 1495)	0.01698 (net 0.01191)
Between *C*. *siamense* and *C*. *gloeosporioides*	3.419 ± 0.1764 (N = 184)	0.03155 (net 0.02539)
Between *C*. *fructicola* and *C*. *gloeosporioides*	3.108 ± 0.132 (N = 520)	0.02857 (net 0.02510)

^1^ The genetic distances here represent the mean ± standard deviation of pairwise nucleotide differences (in percentages, % of nucleotide differences) between pairs of isolates. The percentages of pairwise nucleotide differences between isolates were calculated based on the concatenated sequences of three gene fragments (*ITS*, *CL and GD*, totaling 1362 bp). The strains included in this analysis are shown in Fig 2. While the reference strains for each of the three species shown in Fig 2 were all included but only one representative of each unique multilocus genotype for the new strains obtained in this study was included here. The number of isolates included in the calculations for the three species were 65, 8, and 23 respectively for *C*. *fructicola*, C. *gloeosporioides*, and *C*. *siamense*. N refers to the number of pairwise isolates used to obtain the mean and standard deviations of the genetic distances. The three between species divergences were all significantly greater than those within species divergences at P<0.001.

^2^ the standard nucleotide diversities within and between pairs of species. Values within parenthesis refer to net nucleotide diversity differences between pairs of species.

Due to the small sample sizes of the three species (*C*. *siamense*, *C*. *gloeosporioides*, and *C*. *camelliae*, Table 3), their population genetic analyses were not performed. Thus, in the following population genetic analyses, we focus on *C*. *fructicola*.

### Genetic Variation Within and Among Loci in *C*. *fructicola*

The four sequenced gene fragments differed in their number and percentages of polymorphic nucleotide sites within *C*. *fructicola*. Briefly, in the total sample of 167 isolates, the 539 bp for the *ITS* region contained 19 polymorphic sites (19/539 = 3.52% polymorphic sites). Of the DNA fragments from the three protein-coding genes, the *CL* gene fragment (633 bp) contained four polymorphic sites (= 0.63% polymorphic sites); the *GS* gene fragment (711 bp) contained 60 polymorphic sites (= 8.44% polymorphic sites); and the *GD* gene fragment (190 bp) contained 15 polymorphic sites (= 7.89% polymorphic sites). The total number of polymorphic sites for the combined 2073 bp was 98 (= 4.73% polymorphic sites). Among the four gene fragments, the highest mean nucleotide diversity was found in the *GD* fragment (0.103±0.166; mean ± standard deviation; n = 15), followed by *GS* (0.042±0.066; n = 60), *ITS* (0.024±0.018; n = 19), and *CL* (0.018±0.0068; n = 4). The mean diversity index at the 98 polymorphic nucleotide sites for the combined gene sequences was 0.044±0.009 (n = 98).

Treating each gene fragment as an individual locus, the allele numbers at each of the four loci in the total sample of *C*. *fructicola* are 15, 5, 12, and 11 respectively for the *ITS*, *CL*, *GS*, and *GD* gene fragments. The mean allelic diversity among the four loci for each of the 12 populations with sample sizes greater than 5 is presented in Table 1. The highest mean allelic diversity was found in HNMJH (0.482) and the lowest was found in HNWZS and JXGSA (both with a mean of 0.300) (Table 1). However, due to the large differences in mean allelic diversity among loci within each geographic population, the observed differences among the populations were statistically not significantly different from each other (p>0.05 in all pairwise comparisons, detailed data not shown). The combined mean allelic diversity for all four loci and all populations was 0.377(±0.043).

Interestingly, none of the polymorphisms caused amino acid substitutions (data not shown). All of the observed polymorphic sites in the three protein-coding genes were either in intronic regions or caused synonymous substitutions. The results suggested strong purifying selection at the three protein-coding loci, consistent with their house-keeping functions and essential physiological roles within cells. Similarly, the nucleotide variations observed in the sequenced ITS fragments were found in either ITS1 or ITS2 and no polymorphism was found in the 5.8S rRNA gene located between the ITS1 and ITS2 regions. These results also suggest that the mutations observed here are likely neutral, not involved in geographic or ecological adaptations of the strains.

### Genetic Variation within and between Geographic Populations of *C*. *fructicola*

The summary data for the average number of alleles per locus, the range of allele numbers among the four loci, the total number of private alleles (i.e. allele found only in one plantation) at the four loci, and the number of genotypes within each geographic population of *C*. *fructicola* are presented in Table 1. Aside from the Nanning, Guangxi population where only two isolates of *C*. *fructicola* were obtained and showed no polymorphism, each of the other 14 geographic populations had polymorphism in at least one locus. The largest number of alleles at one locus within an individual plantation was seven, found in two populations (JXCB and HNLY; Table 1), both at locus *GD*. However, these two populations had the largest sample sizes and the large numbers of alleles in these two populations were likely due to their large sample sizes. Indeed, our analyses identified that sample size was positively correlated with: (i) the average number of alleles per locus (Pearson’s correlation coefficient R = 0.8214, p = 0.0003592), (ii) the highest number of alleles at an individual locus (R = 0.9257, p = 1.49e-05), and (iii) the total number of genotypes (R = 0.9427, p = 7.767e-06). However, there was no statistically significant correlation between sample size and the number of private alleles among the 15 geographic populations (R = 0.2574, p = 0.3546).

Sequence variations at the four gene fragments identified a total of 53 multilocus genotypes among the 167 isolates of *C*. *fructicola* (raw genotype data in S2 Table). Of these 53 genotypes, 32 were represented by only one isolate each while the remaining 21 genotypes were each represented by two (9 genotypes) or more (12 genotypes) isolates. Most of these shared genotypes contained strains from multiple plantations and geographic regions. For example, of the 9 genotypes containing two isolates each, six were represented by isolates from different geographic areas. The remaining 12 shared genotypes were each found in at least three tea-oil tree plantations from at least two provinces (S2 Table). The most common genotype (genotype 19 in S2 Table) was represented by 21 isolates from eight tea-oil tree plantations in three provinces/municipality (Jiangxi, Hunan, and Chongqing) (S5 Fig). These results are consistent with broad clonal dispersal abilities of this organism.

### Clonality and Recombination

Our phylogenetic and population genetic tests identified no evidence of recombination among variable nucleotide sites within any of the four sequenced gene fragments (detailed results not shown). This result is consistent with our phylogenetic analysis results. Specifically, in the phylogenetic analyses using the maximum parsimony approach, a single most parsimonious tree was obtained from each gene fragment, suggesting no parallel mutation or recombination within individual genes in our sample. Our results differ from many investigations in bacteria where signatures of intra-genic recombination are commonly observed [26,27,43]. However, our results here are similar to those found in the majority of fungal populations [24,28–30].

Due to the lack of evidence for intra-genic recombination, we used allelic information based on all SNPs at each locus to investigate signatures of recombination between the four gene fragments for the above sample types. Population genetic tests suggested evidence for both clonality and recombination in natural populations of *C*. *fructicola* from tea-oil trees in southern China. Specifically, three pieces of evidence suggested evidence of clonality. First, 21 of the 53 multilocus genotypes were represented by two or more isolates each, with most of these genotypes representing isolates from both the same and different plantations and geographic areas. Second, our phylogenetic compatibility tests indicated that all of our samples had some levels of phylogenetic compatibility (Table 4). Third, in the total sample including all 167 isolates, there was evidence for significant departure from random association of alleles among the four loci (Table 4). We would like to mention that the observed clonality was not due to the lack of sequence variation at the analyzed loci. Specifically, 15, 5, 12, and 11 alleles were found at ITS, CL, GS, and GD loci respectively in our sample of 167 *C*. *fructicola* isolates. Assume random mating, we would expected to have 9900 (15x5x12x11) different genotypes and the chances of finding any two strains with an identical genotype would be extremely small. Thus, we believe that the clonal genotypes observed here reflect at least partly a clonal component of *C*. *fructicola* reproduction in nature.

**Table 4:** Evidence for recombination in natural populations of *Colleotrichum fructicola* infecting leaves of *Camellia oleifera* trees in southern China.

Samples^1^	Sample size	PrPC^2^	rd^3^
All strains	167	0.33	0.053**
Unique genotypes	53	0.33	0.067
Jiangxi Province	49	0.83	0.027
JXGS	25	0.83	0.037
JXGS-A	19	0.83	0.034
JXCB	24	0.83	0.017
Hunan	80	0.5	0.012
HNTJL	12	1.0	0.007
HNMJH	11	0.83	0.056
HNLY	27	0.83	0.010
HNHH	15	0.83	0.002
HBSZ	14	0.83	0.001
FJLY	11	0.83	0.047

^1^ the full names of abbreviated local populations are presented in Table 1.

^2^ PrPC: proportion of phylogenetically compatible pairs of loci. In all cases except Tianjiling in Hunan, there is evidence of phylogenetic incompatibility, consistent with recombination.

^3^ rd, the adjusted index of association (*I*_*A*_,) divided by the number of loci (4);

** P<0.01.

Despite the abundant evidence for clonality, evidence for recombination was also found in our *C*. *fructicola* samples. For example, all individual subpopulations failed to reject the null hypothesis of random recombination (the p values for all I_A_ tests of subpopulations were greater than 0.05). Similarly, all except the Tianjiling population in Hunan (HNTJL), showed evidence of phylogenetic incompatibility (Table 4). Taken together, these results suggest that geographic populations of *C*. *fructicola* from tea-oil trees in southern China contain signatures of both clonality and recombination.

### Relationships among Geographic Populations

**Fig 3:**
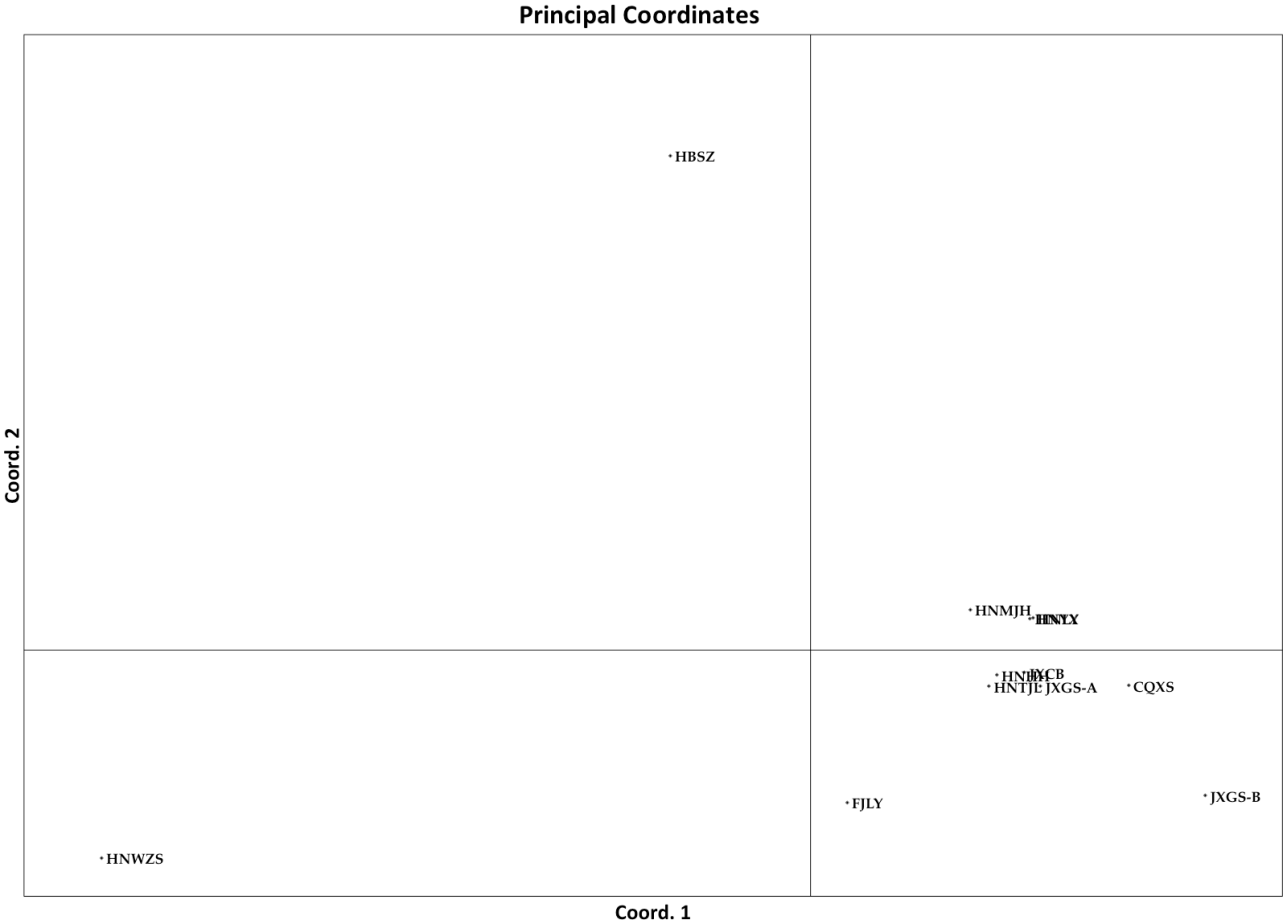
Principal component analyses of the relationships among populations of *Colletotrichum fructicola* from tea-oil trees in southern China. (A) The analysis where 12 geographic populations were included. Populations from three plantations HNWZS, FJLY, and HBSZ showed the biggest difference from populations from the other 10 plantations. (B) The analysis where three populations (HNWZS, HBSZ, and FJLY) were excluded and with only nine populations included.

**Fig 4:**
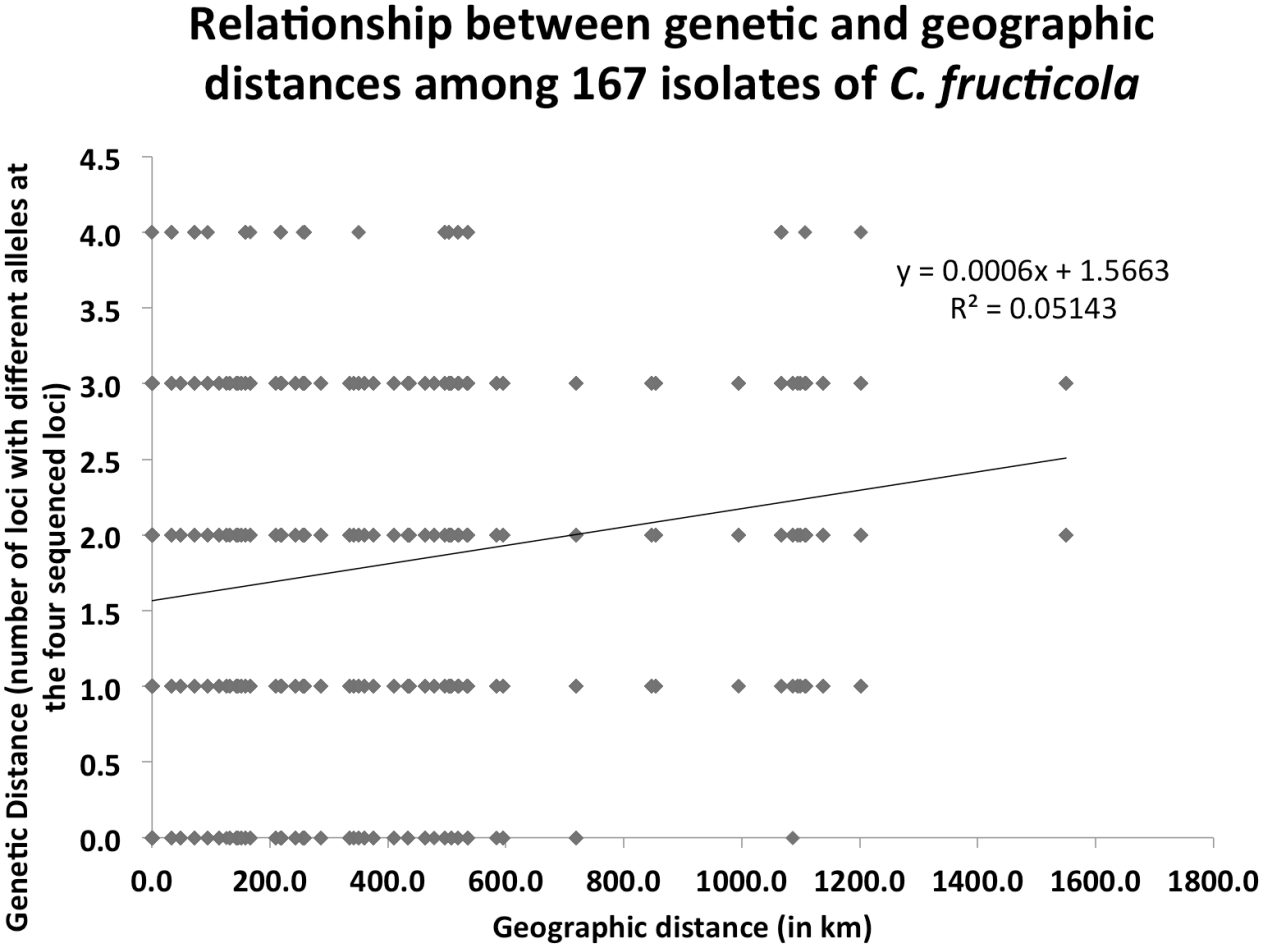
Mantel test result. A Mantel test between genetic distance (allelic differences at four loci, y axis) and geographical distances (in km, calculated based on longitudinal and latitudinal coordinates, x-axis) between all pairs of strains of *Colletotrichum fructicola* from tea-oil trees in southern China (P < 0.05).

Our analyses of molecular variance (AMOVA) of the 12 plantations each with a sample size greater than five indicated that 86% of the observed genetic variations were found within individual plantations and 14% were found among the plantations. The pairwise population F_ST_ values are presented in Table 5. Among the 66 pairwise population comparisons, 41 pairs were significantly differentiated from each other. Three populations, HNWZS, HBSZ, and FJLY, were significantly differentiated from each other and from all other populations (Table 5). This statistically significant contribution of genetic variation from among the plantations (p = 0.01) suggests the existence of gene flow barriers among at least some of the plantations. Results from the principal component analysis (PCA) also indicated that the southernmost (the Wuzhishan population on Hainan Island, HNWZS), the northernmost (the Suizhou population in Hubei province, HBSZ), and the easternmost (the Longyan population in Fujian province, FJLY) were genetically the most distinct among the 12 geographic populations (Fig 3a). Indeed, our Mantel test found that geographic distance was positively correlated to genetic differentiation between pairs of strains in *C*. *fructicola* (p<0.05), with a Pearson’s correlation coefficient of 0.321 (Fig 4).

**Table 5:** Pairwise F_ST_ values between populations of *Colletotrichum fructicola* from 12 different plantations of the tea-oil tree *Camellia oleifera*. Due to the small sample sizes, samples from HNCD, HBXG, and GXNN were not included in the analyses.

	JXGS-B	JXGS-A	JXCB	HNYX	HNWZS	HNTJL	HNMJH	HNLY	HNHH	HBSZ	FJLY
JXGS-A	0.002										
JXCB	0.048	0.000									
HNYX	0.160	0.075	0.097*								
HNWZS	0.455**	0.341***	0.345***	0.358**							
HNTJL	0.112*	0.094**	0.127**	0.033	0.323***						
HNMJH	0.145*	0.081*	0.123*	0.000	0.316**	0.012					
HNLY	0.125*	0.029	0.087**	0.000	0.350***	0.066*	0.000				
HNHH	0.091	0.004	0.016	0.000	0.309***	0.016	0.000	0.000			
HBSZ	0.411***	0.272**	0.251**	0.248**	0.456***	0.284***	0.229**	0.258**	0.240**		
FJLY	0.294**	0.190**	0.120*	0.197*	0.404***	0.235***	0.247**	0.272***	0.128*	0.397**	
CQXS	0.000	0.000	0.062	0.054	0.401***	0.105*	0.080	0.028	0.051	0.334***	0.312**

*, ** and *** represent statistically significant genetic differences between the two populations at P<0.05 (*), P<0.01 (**), and P<0.001 respectively.

Aside from these three geographic populations being genetically very different, several other population pairs also showed some levels of differentiation among each other (Table 5). Indeed, even after populations HBSZ, FJLY, and HNWZS were excluded, the AMOVA results still showed an overall statistically significant genetic differentiation among the remaining nine populations, with 5% of the genetic variation coming from among the populations (P = 0.015) and 95% coming from within populations. However, the genetic differentiation and geographic distance were not significantly correlated among these nine populations (P = 0.390). Fig 3b shows the PCA result after excluding populations HBSZ, FJLY, and HNWZS. Interestingly, the first major axis separated the five populations in Hunan province from the three populations in Jiangxi province and one from Chongqing.

**Fig 5:**
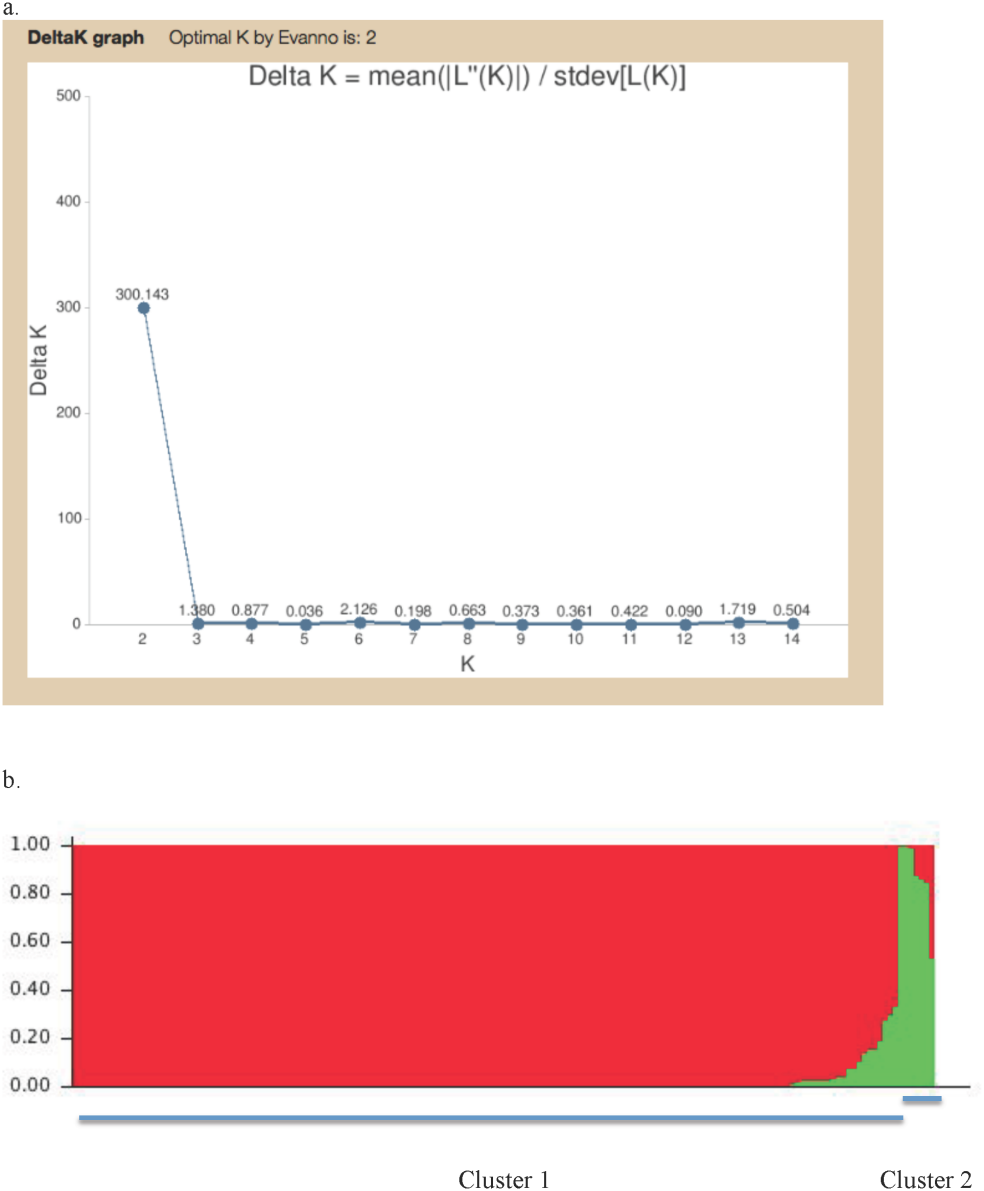
STRUCTURE output. Output from STRUCTURE separating the total *Colletotrichum fructicola* sample into two distinct clusters: (a) The relationship between K and Delta K; and (b) The two major clusters. The presence of intermediate genotypes is consistent with *C*. *fructicola* as a naturally recombining species.

In the STRUCTURE analysis, the information at all 98 polymorphic nucleotide sites for all 167 isolates was included. Our results indicated that the highest-likelihood value and the mode of ΔK index distribution were observed when K = 2 (Fig 5). The K = 2 separated the total sample into one large cluster containing 160 isolates distributed in all 15 geographic populations and a small cluster containing seven isolates from four plantations in four provinces (Fig 5). Specifically, the seven isolates in the second cluster included two from Wuzhishan in Hainan, three from Tianjiling in Hunan, and one each from Guangshang in Jiangxi and Xiaogang in Hubei. The results are also consistent with the phylogenetic analyses results of all 167 *C*. *fructicola* isolates based on four loci as shown in S5 Fig and the species identification results based on three loci shown in Fig 2. STRUCTURE analysis using clone-corrected genotype data identified the same pattern as that from using all strains without clone correction (Result not shown).

As described in Materials and Methods, two tests were used to investigate whether the two tea-oil tree breeding centers JXCB and HNTJL might also contain the highest genetic diversities of *C*. *fructicola* and potentially serve as the distribution centers of the *C*. *fructicola* pathogen genetic variation. In the first set of tests, the two populations of C. fructicola from the tea-oil tree breeding centers HNTJL and JXCB did not show statistically significant higher number of alleles or higher allelic diversities than other populations (Table 1, detailed results not shown). However, the second test using the DIYABC program suggested that the first scenario (i.e. the *C*. *fructicola* samples from the two tea-oil tree breeding centers diverged earlier than those from other plantations) was more likely than the second scenario (i.e. all the population samples diverged at the same time) (S3 Table). Our results are consistent with the hypothesis that the two tea-oil tree breeding centers likely contribute to *C*. *fructicola* pathogen genetic diversity in commercial plantations.

## Discussions

In this study, we obtained and analyzed the patterns of genetic variation among isolates and populations of CGSC from tea-oil tree leaves with anthracnose syndromes in southern China. A total of 199 isolates belonging to the CGSC were obtained from 15 tea-oil tree plantations in seven provinces, representing the broad geographic distribution of tea-oil tree plantations in China. The relatively low isolation rates of CGSC among many of our samples may be caused by several factors, including the stringent disinfection procedure used to kill leaf surface microbes, the different severity of leaf infection (thus the original fungal load in the leaves), the application of fungicides in some of the plantations, the delay between field sampling and lab isolation, and/or the presence of other pathogens causing similar symptoms as CGSC. Sequence analyses of four gene fragments identified that the 199 isolates clustered with strains of four known species. However, five of the isolates did not have clear species affiliations. The dominant species was *C*. *fructicola*, represented by 167 isolates distributed in all 15 plantations. The 167 isolates belonged to 53 multilocus sequence types, among which 21 were each represented by two or more isolates. Eighteen of the 21 genotypes were distributed in at least two provinces, consistent with clonal dispersals among plantations and geographic populations. However, our analyses also identified evidence for recombination in all regional and local populations, consistent with sexual reproduction of this organism in nature. Though the majority of genetic variation was found within local populations, geographic populations showed evidence for significant genetic differentiation. Specifically, the northernmost (HBSZ), easternmost (FJLY) and southernmost (HNWZS) populations were significant different from each other and from other geographic populations. Furthermore, there is genetic evidence that the two tea-oil tree breeding centers might have contributed to *C*. *fructicola* pathogen diversity in commercial tea-oil tree plantations. Below we discuss the limitations of our results and the implications of our study on the epidemiology and management of anthracnose diseases on tea-oil trees.

### Species Distribution

Following criteria established previously [7,11], our results identified that of the 199 isolates of CGSC obtained here, the majority were *C*. *fructicolca* (167 isolates, 84%). Our result differs from those reported previously on the causative agents of anthracnose on tea-oil trees. Previous studies have reported *C*. *gloeosporioides* as the causal agent [10,12]. However, most such studies relied only on morphological features and/or sequences at only one locus (usually ITS) for identification. As has been indicated recently [7,11], morphological features alone are insufficient to distinguish species within CGSC. In addition, there are significant problems in the GenBank database with regard to the accuracy of fungal ITS sequences [50], including those in CGSC [5]. As a result, identification based only on BLAST comparisons of ITS sequences with GenBank accessions can be unreliable and misleading. In this study, we used reference sequences at multiple loci from the type, ex-type or confirmed voucher specimens of CGSC [6,7,51] for our identifications. In addition, our study is the first to use multiple gene sequences to analyze a large sample of CGSC from anthracnose-infected leaves of tea-oil trees. Our results revealed that only six of the 199 strains belonged to the *C*. *gloeosporioides* sensu stricto. This result is similar to what was recently reported for anthracnose causal agents of tropical fruits [8]. In their analysis of sequence data at five gene fragments, none of their 25 isolates from eight tropical fruits were *C*. *gloeosporioides* sensu stricto and two strains were identified as belonging to *C*. *fructicola* [8]. Taken together, these results highlight the importance of using multiple gene sequences and sequences from confirmed reference specimens for species identifications within the *C*. *gloeosporioides* species complex. Interestingly, five of the 199 isolates had no clear phylogenetic affiliations with the 22 known species [7,11]. Additional genetic and phenotypic information are needed in order to determine their taxonomic status.

### Genetic Variation and Mode of Reproduction Of *C*. *fructicola*

Our analyses identified that the four analyzed gene fragments were polymorphic in most local populations of *C*. *fructicola* from tea-oil trees in southern China. Of the four gene fragments, GS was found to contain the highest proportion of polymorphic nucleotide sites, followed by GD, ITS, and CD. The total number of alleles per locus ranged from 5 to 15 in our *C*. *fructicola* sample. Interestingly, despite the extensive polymorphisms within each locus, we found no evidence of heterozygosity within any of the isolates at these loci, consistent with the haploid status of this species in nature. These results support previous conclusions that these four loci are excellent markers for identifying genotypes of *C*. *fructicola*.

As expected, evidence for both clonality and recombination were found for most geographic populations of *C*. *fructicola*. The results here are similar to most fungal populations in nature [25,26,29,38,39,51,52]. However, on the broader taxonomic scale of CGSC, there is very limited information about the relationship between the sexual teleomorph(s) collectively called *Glomerella cingulata* and the 22 known phylogenetic species within CGSC defined based on gene sequences. For example, we don’t know whether each phylogenetic species within CGSC has a distinct corresponding sexual state in nature. At present, only three of the 22 phylogenetic species have been reported to have corresponding teleomorphs in nature: *C*. *fructicola* (corresponding to *Glomerella cingulata* var. *minor*); *C*. *kahawae* (corresponding to *Glomerella cingulata* var. *migrans*); and *C*. *camelliae* (corresponding to *Glomerella cingulata* f. sp. *camelliae*) [7,31,32]. In contrast, the sexual state for other 19 phylogenetic species have not been reported. However, whether sexual reproduction exists in nature in individual species can be inferred using data from population genetic analyses [25,26,29,39,53]. As shown in this study, our population genetic analyses identified evidence for recombination in *C*. *fructicola*, consistent with the expectation of mating and sexual reproduction in nature for this species [7,32]. Our results suggest that a similar approach can be used to determine whether sexual cycles exist in other phylogenetic species of CGSC without the need to directly observe their sexual reproductive structures in nature.

### Genetic Variation Among Geographic Populations

Our study identified a statistically significant contribution of geographic separation to the overall observed genetic variation within *C*. *fructicola* from tea-oil tree leaves. Among the 66 pairwise population comparisons, 41 rejected the null hypothesis of no significant genetic differences while the remaining 25 failed to reject the null hypothesis. Some of the 25 pairwise comparisons that failed to reject the null hypothesis were likely due to their small sample sizes and not a true lack of differences. For example, samples from JXCB-B and HNYX had only six isolates each. Even though the F_ST_ value was relatively high (at 0.16, Table 5), the p value was 0.079. Doubling the sample sizes for both populations while maintaining the same gene and genotype frequencies would increase the F_ST_ value and result in an P value 0.002, rejecting the null hypothesis (S6 Fig). Alternatively, at one locus with two alleles, two samples with six isolates each can only show a statistically significant difference when the F_ST_ value is 1 (i.e. when the two samples are fixed for different alleles). Taken together, the observed genetic differentiation among geographic populations in our study was likely an underestimate of the true genetic differentiations among these populations in nature.

The observed genetic differentiation among geographic populations could be due to (i) gene flow barriers, (ii) adaptations to local ecological niches, (iii) differential clonal expansions of different genotypes at different locations, or (iv) combinations of the aforementioned three factors. The wide geographic distributions of many multilocus genotypes suggest that geographic barriers were unlikely solely responsible for the genetic differentiation among geographic populations of *C*. *fructicola*. Instead, local selection and adaptation as well as local clonal expansion might have been important factors. A recent study of geographic populations of another anthracnose-causing fungal pathogen *Colletotrichum truncatum* from chili peppers in China also identified a significant contribution of the north-south divide to the observed genetic variations [40]. Given the broad germplasm exchanges and frequent commercial chili pepper trade among regions within China, the authors suggested that differences in climate and other ecological factors were likely responsible for their observations. Indeed, anthropogenic activities have accelerated the emergence and dispersals of many plant fungal pathogens [30,38,40,53].

At present, the specific ecological factors that might have contributed to the genetic differentiation among local *C*. *fructicola* populations from tea-oil trees are not known. However, abiotic factors such as temperature and biotic factors such as host trees as well as populations of *C*. *fructicola* from other host plants in the vicinity of the sampled tea-oil tree plantations could have all contributed to genetic differentiations. For example, other trees as well as crops such as peanuts, watermelon, and chili peppers are often grown in or around the tea-oil tree plantations and some of these plants can also suffer anthracnose diseases. At present, their causative agents and epidemiology are largely unknown. It’s entirely possible that cross-infection and recombination can occur between pathogens from different host plants, contributing to the genetic differences among the plantations. In addition, latitudinal differences could have contributed to adaptations to different temperatures between the southern-most and northern-most populations here, as was suggested for *C*. *truncatum* [40]. For example, Wuzishan in Hainan (HNWZS) has a tropical climate with an annual average temperature of ~25°C and while Shuizhou in Hubei (HBSZ) has an annual average temperature of ~16°C. The large temperature differences could select for genotypes that are locally adapted to their specific conditions.

Previous studies of plant fungal pathogens suggested that the centers of host origin and/or host diversity were often associated with greater genetic diversity of fungal pathogens, including greater allelic diversity, higher genotypic diversity, and/or more private alleles [1–3,21,38,40,53]. The results from our two tests suggested slightly different conclusions about the effects of host diversity on pathogen diversity. In the first test, the two germplasm centers (JXCB and HNTJL) of tea-oil trees since the 1960s did not show significantly higher allelic or genotypic diversities than the remaining sampled sites that had only one to a few cultivars at each site. For example, neither the JXCB nor the HNTJL had the highest allelic diversity among the plantations. Even though the HNTJL plantation contained the highest number of private alleles at the four sequenced loci, the other host diversity center (Changbu in Jiangxi, JXCB) had a low number of private alleles, despite a large sample size from this population (24, twice that of HNTJL). In contrast to results from the first tests, the second test using DIYABC suggested that the two breeding centers likely contributed to the genetic diversities in other plantations. Given the conflicting results between the two tests, we believe caution should be taken in interpreting the DIYABC results. More extensive sampling should be made from most of the plantations before a firm conclusion could be drawn.

The second biotic factor that could have influenced the local *C*. *fructicola* genetic diversity in tea-oil trees is the populations of *C*. *fructicola* from crops and trees in fields and forests adjacent to the tea-oil tree plantations. Among the 15 tea-oil tree plantations, the Wuzhishan plantation from Hainan Island was by far the youngest (only four years old at the time of sampling in 2012). The tea-oil trees in this plantation were imported as seedlings from the Changbu plantation in Jiangxi province (JXCB) in south-central China. Interestingly, despite evidence for clonal genotype dispersal among other geographic areas, none of the five *C*. *fructicola* isolates from this plantation had a genotype identical to those from the Changbu plantation in Jiangxi (or any other population in Mainland China). Instead, all five isolates from the HNWZS plantation had the same allele at the *GD* locus and this allele was not found in Changbu, Jiangxi (S2 Table). However, alleles at loci *ITS* and *CL* from isolates in Wuzhishan were found in Changbu and several other locations (S2 Table). Four of the five strains from Wuzhishan also contained a unique private allele at the *GS* locus not found in any other isolates. These results suggest that genotypes of *C*. *fructicola* infecting the Wuzhishan tea-oil trees may represent host shifts of native *C*. *fructicola* genotypes from other hosts in Hainan to the new tea-oil trees in the sampled plantation. Indeed, host-jump has shown to accelerate ecological speciation of a closely related species in CGSC, *Colletotrichum kahawae* [30]. Alternatively, they may also represent recombinant genotypes between native isolates and migrants from Changbu, Jiangxi (JXCB). As demonstrated by the DIYABC analyses, there is some statistical support for the JXCB sample being one of the earlier divergent populations of *C*. *fructicola*. In addition, hybridization has been found in natural populations of a diversity of fungi [1,2,28,29,54]. *C*. *fructicola* is known to have broad host ranges and can undergo sexual mating [7,31]. More strains from a diversity of host plants in Hainan are needed in order to identify the potential origin(s) of these genotypes on Hainan Island.

Despite the observed geographic differentiation among local populations, STRUCTURE analyses showed that most *C*. *fructicola* isolates (160/167) from all 15 plantations analyzed here belonged to one large interbreeding population. The seemingly contradictory results were likely due to the different assumptions and methods of the two analyses (i.e. STRUCTURE and AMOVA) [44–47]. For example, AMOVA analyzes the differences in allele frequencies within and between pre-defined populations and partition the total genetic variation to different hierarchical levels based on geography or other factors. In contrast, STRUCTURE analyzes the relationships among alleles to partition natural strains into inter-breeding groups. In the presence of differential clonal propagations of different genotypes within and among geographic areas, AMOVA could reveal significant differentiations among the populations while STRUCTURE may identify a single interbreeding population due to the random associations of alleles. Interestingly, the existence of a distinct cluster containing five genotypes distributed in four plantations in four provinces suggests that *C*. *fructicola* from tea-oil tree may contain two cryptic species. The analyses of more strains from these plantations and additional phenotypic and genotypic characterizations should help reveal the taxonomic relationships between these two clusters.

### Conclusions and Perspectives

This study revealed high genetic variation, clonal genotype dispersion, limited but unambiguous evidence for recombination within and among geographic populations of *C*. *fructicola* on tea-oil tree leaves in southern China. Our results provide a foundation from which to further explore the epidemiology and management strategies of tea-oil tree anthracnose in southern China. However, additional information will be needed in order to develop effective strategies for managing anthracnose in tea-oil tree plantations. For example, one essential piece of information would be the specificity of host-pathogen interaction between the cultivars of tea-oil plants and species of CGSC and genotypes of *C*. *fructicola*. Hundreds of tea-oil tree cultivars have been officially registered [21]. However, most such cultivars have not been genetically defined and the genetic relationships among the majority of these cultivars remain unknown. As a result, nothing is known about the relationship between host tree genotypes and CGSC pathogen genotypes in nature.

Similarly, very little is known about the patterns of genetic variation among samples of *C*. *fructicola* from other tissues and organs of tea-oil plants as well as from other species of plants. If similar genotypes were found among *C*. *fructicola* samples from other plants growing nearby, a more holistic approach would be needed to take into account of the life histories of different hosts in order to develop effective strategies for controlling anthracnose. Our study provides a foundation from which to investigate these and other related issues.

## Supporting Information

S1 Fig Neighbour-joining tree of the unique ITS sequences from each geographic location in our sample and their relationships to those closely related from the GenBank. Numbers in parenthesis indicate the number of strains with that sequence type from the specific plantations.
(TIFF)

S2 Fig Neighbour-joining tree of the unique Calmodulin (CL) sequences from each geographic location in our sample. Numbers in parenthesis indicate the number of strains with that sequence type from the specific plantation.
(TIFF)

S3 Fig Neighbour-joining tree of the unique glutamine synthetase (GS) sequences from each geographic location in our sample. Numbers in parenthesis indicate the number of strains with that sequence type from the specific plantation.
(TIFF)

S4 Fig Neighbour-joining tree of the unique glyceraldehyde-3-phosphate dehydrogenase (GD) sequences from each geographic location in our sample. Numbers in parenthesis indicate the number of strains with that sequence type from the specific plantation.
(TIFF)

S5 Fig Neighbor-Joining phylogeny showing the relationships among the 167 *C*. *fructicola* strains based on sequences at four gene loci. (TIFF)

S6 Fig An example of the effects of changing sample size on F_ST_ values between samples JXGS-B and HNLY. (A) When the sample sizes were six at each of the two populations as we currently have; and (B) When the sample sizes were doubled to 12 each.
(TIFF)

S1 Table GenBank accession numbers for all 796 sequences obtained in this study. (DOCX)

S2 Table Allelic and genotype data for all 167 isolates of *C*. *fructicola* from southern China. Highlighted samples were excluded from population genetic analyses. For each isolate, the string of capital letters indicates its geographic location corresponding to those in Table 1.
(DOCX)

S3 Table DIYABC output with regard to the two competing scenarios of population history. (DOCX)
