# Supplementary figures and images for: Population Genetic Analyses of the Fungal Pathogen Colletotrichum fructicola on Tea-Oil Trees in China
Source: PLoS One. 2016 Jun 14;11(6):e0156841. doi: 10.1371/journal.pone.0156841 (PMC4907445; doi:10.1371/journal.pone.0156841)

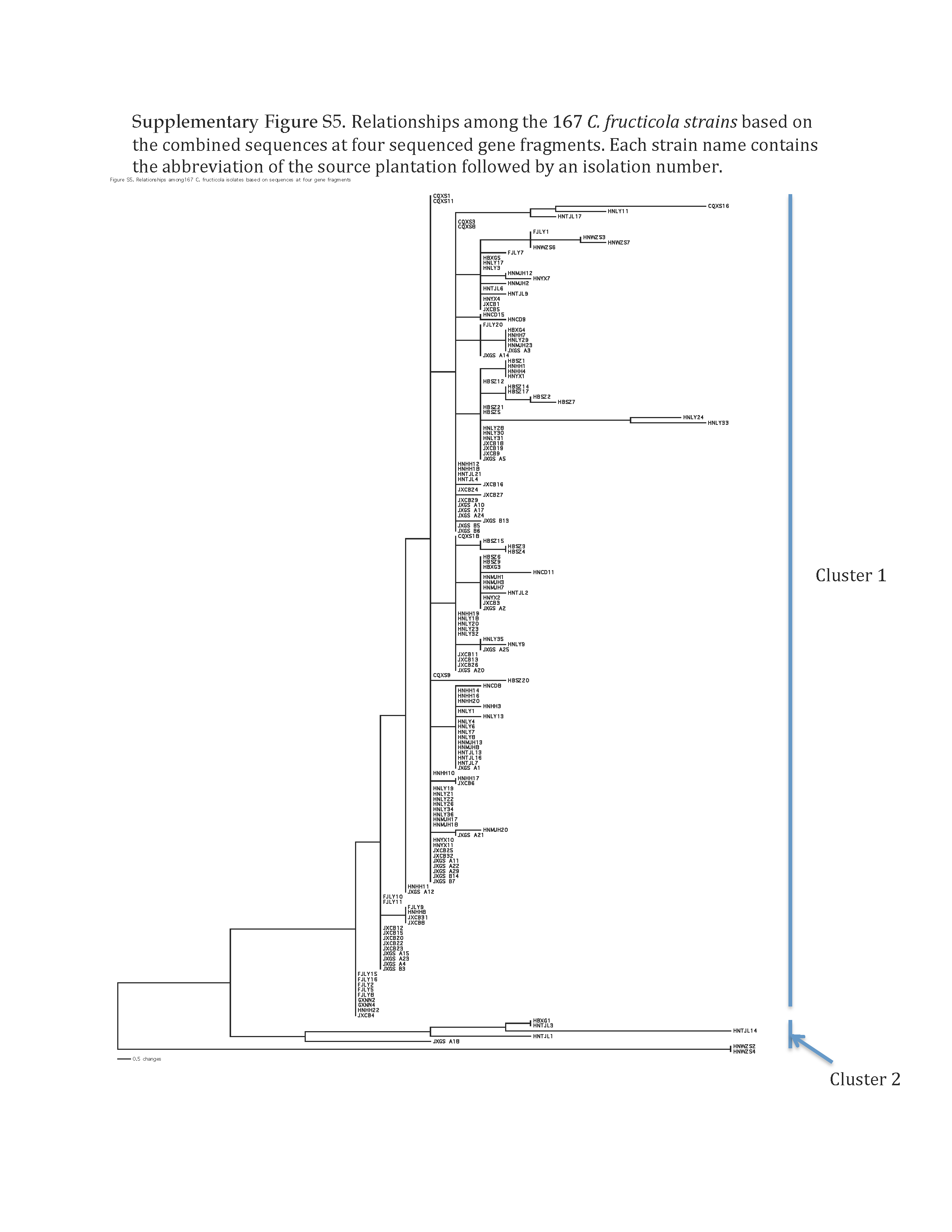

Supplement: S5 Fig — (TIFF) [file pone.0156841.s005.tiff]

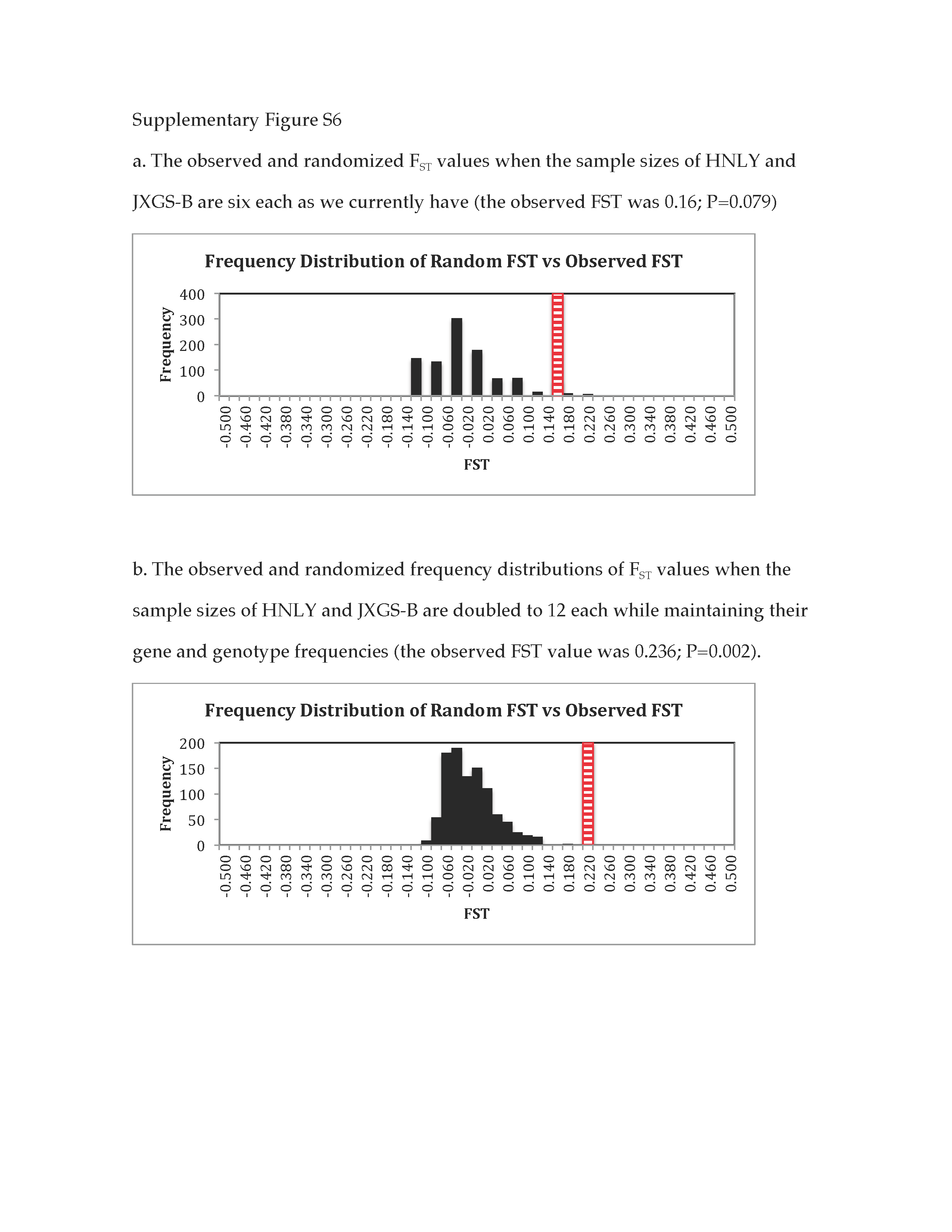

Supplement: S6 Fig — (A) When the sample sizes were six at each of the two populations as we currently have; and (B) When the sample sizes were doubled to 12 each. (TIFF) [file pone.0156841.s006.tiff]
