# Supplementary material for: Population Genetic Analyses of the Fungal Pathogen Colletotrichum fructicola on Tea-Oil Trees in China
Source: PLoS One. 2016 Jun 14;11(6):e0156841. doi: 10.1371/journal.pone.0156841 (PMC4907445; doi:10.1371/journal.pone.0156841)
Supplement: S1 Table — (DOCX) [file pone.0156841.s007.docx]

S1 Table. GenBank accession numbers for sequences of the 199 isolates obtained in this study.

| **Isolate designation** | **Taxon** | **CAL** | **GS** | **GD** | **ITS** |
| --- | --- | --- | --- | --- | --- |
| CQXS16 | *C. fructicola* | KJ132224 | KJ132025 | KJ131826 | KJ131627 |
| CQXS1 | *C. fructicola* | KJ132219 | KJ132020 | KJ131821 | KJ131622 |
| CQXS11 | *C. fructicola* | KJ132223 | KJ132024 | KJ131825 | KJ131626 |
| CQXS4 | [*Colletotrichum* sp.](http://www.ncbi.nlm.nih.gov/nuccore/KJ132176.1) | KJ132176 | KJ131977 | KJ131778 | KJ131579 |
| CQXS8 | *C. fructicola* | KJ132221 | KJ132022 | KJ131823 | KJ131624 |
| CQXS9 | *C. fructicola* | KJ132222 | KJ132023 | KJ131824 | KJ131625 |
| CQXS3 | *C. fructicola* | KJ132220 | KJ132021 | KJ131822 | KJ131623 |
| CQXS18 | *C. fructicola* | KJ132225 | KJ132026 | KJ131827 | KJ131628 |
| HBSZ1 | *C. fructicola* | KJ132226 | KJ132027 | KJ131828 | KJ131629 |
| HBSZ2 | *C. fructicola* | KJ132227 | KJ132028 | KJ131829 | KJ131630 |
| HBSZ3 | *C. fructicola* | KJ132228 | KJ132029 | KJ131830 | KJ131631 |
| HBSZ4 | *C. fructicola* | KJ132229 | KJ132030 | KJ131831 | KJ131632 |
| HBSZ5 | *C. fructicola* | KJ132230 | KJ132031 | KJ131832 | KJ131633 |
| HBSZ6 | *C. fructicola* | KJ132231 | KJ132032 | KJ131833 | KJ131634 |
| HBSZ7 | *C. fructicola* | KJ132232 | KJ132033 | KJ131834 | KJ131635 |
| HBSZ9 | *C. fructicola* | KJ132233 | KJ132034 | KJ131835 | KJ131636 |
| HBSZ12 | *C. fructicola* | KJ132234 | KJ132035 | KJ131836 | KJ131637 |
| HBSZ14 | *C. fructicola* | KJ132235 | KJ132036 | KJ131837 | KJ131638 |
| HBSZ15 | *C. fructicola* | KJ132236 | KJ132037 | KJ131838 | KJ131639 |
| HBSZ17 | *C. fructicola* | KJ132237 | KJ132038 | KJ131839 | KJ131640 |
| HBSZ20 | *C. fructicola* | KJ132238 | KJ132039 | KJ131840 | KJ131641 |
| HBSZ21 | *C. fructicola* | KJ132239 | KJ132040 | KJ131841 | KJ131642 |
| HBXG1 | *C. fructicola* | KJ132240 | KJ132041 | KJ131842 | KJ131643 |
| HBXG3 | *C. fructicola* | KJ132241 | KJ132042 | KJ131843 | KJ131644 |
| HBXG4 | *C. fructicola* | KJ132242 | KJ132043 | KJ131844 | KJ131645 |
| HBXG5 | *C. fructicola* | KJ132243 | KJ132044 | KJ131845 | KJ131646 |
| JXGS-A1 | *C. fructicola* | KJ132244 | KJ132045 | KJ131846 | KJ131647 |
| JXGS-A2 | *C. fructicola* | KJ132245 | KJ132046 | KJ131847 | KJ131648 |
| JXGS-A3 | *C. fructicola* | KJ132246 | KJ132047 | KJ131848 | KJ131649 |
| JXGS-A4 | *C. fructicola* | KJ132247 | KJ132048 | KJ131849 | KJ131650 |
| JXGS-A5 | *C. fructicola* | KJ132248 | KJ132049 | KJ131850 | KJ131651 |
| JXGS-A10 | *C. fructicola* | KJ132249 | KJ132050 | KJ131851 | KJ131652 |
| JXGS-A11 | *C. fructicola* | KJ132250 | KJ132051 | KJ131852 | KJ131653 |
| JXGS-A12 | *C. fructicola* | KJ132251 | KJ132052 | KJ131853 | KJ131654 |
| JXGS-A14 | *C. fructicola* | KJ132252 | KJ132053 | KJ131854 | KJ131655 |
| JXGS-A15 | *C. fructicola* | KJ132253 | KJ132054 | KJ131855 | KJ131656 |
| JXGS-A16 | *C. siamense* | KJ132183 | KJ131984 | KJ131785 | KJ131586 |
| JXGS-A17 | *C. fructicola* | KJ132254 | KJ132055 | KJ131856 | KJ131657 |
| JXGS-A18 | *C. fructicola* | KJ132255 | KJ132056 | KJ131857 | KJ131658 |
| JXGS-A20 | *C. fructicola* | KJ132256 | KJ132057 | KJ131858 | KJ131659 |
| JXGS-A21 | *C. fructicola* | KJ132257 | KJ132058 | KJ131859 | KJ131660 |
| JXGS-A22 | *C. fructicola* | KJ132258 | KJ132059 | KJ131860 | KJ131661 |
| JXGS-A23 | *C. fructicola* | KJ132259 | KJ132060 | KJ131861 | KJ131662 |
| JXGS-A24 | *C. fructicola* | KJ132260 | KJ132061 | KJ131862 | KJ131663 |
| JXGS-A25 | *C. fructicola* | KJ132261 | KJ132062 | KJ131863 | KJ131664 |
| JXGS-A28 | [*Colletotrichum* sp.](http://www.ncbi.nlm.nih.gov/nuccore/KJ132176.1) | KJ132178 | KJ1311979 | KJ131780 | KJ131581 |
| JXGS-A29 | *C. fructicola* | KJ132262 | KJ132063 | KJ131864 | KJ131665 |
| JXGS-B3 | *C. fructicola* | KJ132263 | KJ132064 | KJ131865 | KJ131666 |
| JXGS-B5 | *C. fructicola* | KJ132264 | KJ132065 | KJ131866 | KJ131667 |
| JXGS-B6 | *C. fructicola* | KJ132265 | KJ132066 | KJ131867 | KJ131668 |
| JXGS-B7 | *C. fructicola* | KJ132266 | KJ132067 | KJ131868 | KJ131669 |
| JXGS-B8 | *C. camelliae* | KJ132181 | KJ131982 | KJ131783 | KJ131584 |
| JXGS-B11 | [*Colletotrichum* sp.](http://www.ncbi.nlm.nih.gov/nuccore/KJ132176.1) | KJ132177 | KJ1311978 | KJ131779 | KJ131580 |
| JXGS-B12 | [*Colletotrichum* sp.](http://www.ncbi.nlm.nih.gov/nuccore/KJ132176.1) | KJ132179 | KJ1311980 | KJ131781 | KJ131582 |
| JXGS-B13 | *C. fructicola* | KJ132267 | KJ132068 | KJ131869 | KJ131670 |
| JXGS-B14 | *C. fructicola* | KJ132268 | KJ132069 | KJ131870 | KJ131671 |
| JXCB1 | *C. fructicola* | KJ132269 | KJ132070 | KJ131871 | KJ131672 |
| JXCB2 | *C. siamense* | KJ132182 | KJ131983 | KJ131784 | KJ131585 |
| JXCB3 | *C. fructicola* | KJ132270 | KJ132071 | KJ131872 | KJ131673 |
| JXCB4 | *C. fructicola* | KJ132271 | KJ132072 | KJ131873 | KJ131674 |
| JXCB5 | *C. fructicola* | KJ132272 | KJ132073 | KJ131874 | KJ131675 |
| JXCB6 | *C. fructicola* | KJ132273 | KJ132074 | KJ131875 | KJ131676 |
| JXCB8 | *C. fructicola* | KJ132274 | KJ132075 | KJ131876 | KJ131677 |
| JXCB9 | *C. fructicola* | KJ132275 | KJ132076 | KJ131877 | KJ131678 |
| JXCB11 | *C. fructicola* | KJ132276 | KJ132077 | KJ131878 | KJ131679 |
| JXCB12 | *C. fructicola* | KJ132277 | KJ132078 | KJ131879 | KJ131680 |
| JXCB13 | *C. fructicola* | KJ132278 | KJ132079 | KJ131880 | KJ131681 |
| JXCB15 | *C. fructicola* | KJ132279 | KJ132080 | KJ131881 | KJ131682 |
| JXCB16 | *C. fructicola* | KJ132280 | KJ132081 | KJ131882 | KJ131683 |
| JXCB18 | *C. fructicola* | KJ132281 | KJ132082 | KJ131883 | KJ131684 |
| JXCB19 | *C. fructicola* | KJ132282 | KJ132083 | KJ131884 | KJ131685 |
| JXCB20 | *C. fructicola* | KJ132283 | KJ132084 | KJ131885 | KJ131686 |
| JXCB21 | *C. camelliae* | KJ132180 | KJ131981 | KJ131782 | KJ131583 |
| JXCB22 | *C. fructicola* | KJ132284 | KJ132085 | KJ131886 | KJ131687 |
| JXCB23 | *C. fructicola* | KJ132285 | KJ132086 | KJ131887 | KJ131688 |
| JXCB24 | *C. fructicola* | KJ132286 | KJ132087 | KJ131888 | KJ131689 |
| JXCB25 | *C. fructicola* | KJ132287 | KJ132088 | KJ131889 | KJ131690 |
| JXCB26 | *C. fructicola* | KJ132288 | KJ132089 | KJ131890 | KJ131691 |
| JXCB27 | *C. fructicola* | KJ132289 | KJ132090 | KJ131891 | KJ131692 |
| JXCB29 | *C. fructicola* | KJ132290 | KJ132091 | KJ131892 | KJ131693 |
| JXCB31 | *C. fructicola* | KJ132291 | KJ132092 | KJ131893 | KJ131694 |
| JXCB32 | *C. fructicola* | KJ132292 | KJ132093 | KJ131894 | KJ131695 |
| HNTJL1 | *C. fructicola* | KJ132293 | KJ132094 | KJ131895 | KJ131696 |
| HNTJL2 | *C. fructicola* | KJ132294 | KJ132095 | KJ131896 | KJ131697 |
| HNTJL3 | *C. fructicola* | KJ132295 | KJ132096 | KJ131897 | KJ131698 |
| HNTJL4 | *C. fructicola* | KJ132296 | KJ132097 | KJ131898 | KJ131699 |
| HNTJL6 | *C. fructicola* | KJ132297 | KJ132098 | KJ131899 | KJ131700 |
| HNTJL7 | *C. fructicola* | KJ132298 | KJ132099 | KJ131900 | KJ131701 |
| HNTJL8 | *C. siamense* | KJ132198 | KJ131999 | KJ131800 | KJ131601 |
| HNTJL9 | *C. fructicola* | KJ132299 | KJ132100 | KJ131901 | KJ131702 |
| HNTJL10 | *C. siamense* | KJ132199 | KJ132000 | KJ131801 | KJ131602 |
| HNTJL12 | *C. siamense* | KJ132189 | KJ131990 | KJ131791 | KJ131592 |
| HNTJL13 | *C. fructicola* | KJ132300 | KJ132101 | KJ131902 | KJ131703 |
| HNTJL14 | *C. fructicola* | KJ132301 | KJ132102 | KJ131903 | KJ131704 |
| HNTJL16 | *C. fructicola* | KJ132302 | KJ132103 | KJ131904 | KJ131705 |
| HNTJL17 | *C. fructicola* | KJ132303 | KJ132104 | KJ131905 | KJ131706 |
| HNTJL18 | *C. siamense* | KJ132188 | KJ131989 | KJ131790 | KJ131591 |
| HNTJL19 | *C. siamense* | KJ132191 | KJ131992 | KJ131793 | KJ131594 |
| HNTJL20 | *C. siamense* | KJ132200 | KJ132001 | KJ131802 | KJ131603 |
| HNTJL21 | *C. fructicola* | KJ132304 | KJ132105 | KJ131906 | KJ131707 |
| HNTJL22 | *C. siamense* | KJ132194 | KJ131995 | KJ131796 | KJ131597 |
| HNTJL23 | *C. siamense* | KJ132195 | KJ131996 | KJ131797 | KJ131598 |
| HNMJH1 | *C. fructicola* | KJ132305 | KJ132106 | KJ131907 | KJ131708 |
| HNMJH2 | *C. fructicola* | KJ132306 | KJ132107 | KJ131908 | KJ131709 |
| HNMJH3 | *C. fructicola* | KJ132307 | KJ132108 | KJ131909 | KJ131710 |
| HNMJH7 | *C. fructicola* | KJ132308 | KJ132109 | KJ131910 | KJ131711 |
| HNMJH8 | *C. fructicola* | KJ132309 | KJ132110 | KJ131911 | KJ131712 |
| HNMJH9 | *C. siamense* | KJ132186 | KJ131987 | KJ131788 | KJ131589 |
| HNMJH10 | *C. siamense* | KJ132196 | KJ131997 | KJ131798 | KJ131599 |
| HNMJH11 | *C. siamense* | KJ132187 | KJ131988 | KJ131789 | KJ131590 |
| HNMJH12 | *C. fructicola* | KJ132310 | KJ132111 | KJ131912 | KJ131713 |
| HNMJH13 | *C. fructicola* | KJ132311 | KJ132112 | KJ131913 | KJ131714 |
| HNMJH16 | *C. siamense* | KJ132184 | KJ131985 | KJ131786 | KJ131587 |
| HNMJH17 | *C. fructicola* | KJ132312 | KJ132113 | KJ131914 | KJ131715 |
| HNMJH18 | *C. fructicola* | KJ132313 | KJ132114 | KJ131915 | KJ131716 |
| HNMJH20 | *C. fructicola* | KJ132314 | KJ132115 | KJ131916 | KJ131717 |
| HNMJH23 | *C. fructicola* | KJ132315 | KJ132116 | KJ131917 | KJ131718 |
| HNYX1 | *C. fructicola* | KJ132316 | KJ132117 | KJ131918 | KJ131719 |
| HNYX2 | *C. fructicola* | KJ132317 | KJ132118 | KJ131919 | KJ131720 |
| HNYX4 | *C. fructicola* | KJ132318 | KJ132119 | KJ131920 | KJ131721 |
| HNYX7 | *C. fructicola* | KJ132319 | KJ132120 | KJ131921 | KJ131722 |
| HNYX10 | *C. fructicola* | KJ132320 | KJ132121 | KJ131922 | KJ131723 |
| HNYX11 | *C. fructicola* | KJ132321 | KJ132122 | KJ131923 | KJ131724 |
| HNCD10 | *C. siamense* | KJ132190 | KJ131991 | KJ131792 | KJ131593 |
| HNCD2 | *C. gloeosporides* | KJ132169 | KJ131970 | KJ131771 | KJ131572 |
| HNCD4 | *C. gloeosporides* | KJ132170 | KJ131971 | KJ131772 | KJ131573 |
| HNCD8 | *C. fructicola* | KJ132322 | KJ132123 | KJ131924 | KJ131725 |
| HNCD9 | *C. fructicola* | KJ132323 | KJ132124 | KJ131925 | KJ131726 |
| HNCD11 | *C. fructicola* | KJ132324 | KJ132125 | KJ131926 | KJ131727 |
| HNCD12 | *C. gloeosporides* | KJ132171 | KJ131972 | KJ131773 | KJ131574 |
| HNCD13 | *C. gloeosporides* | KJ132172 | KJ131973 | KJ131774 | KJ131575 |
| HNCD14 | *C. gloeosporides* | KJ132173 | KJ131974 | KJ131775 | KJ131576 |
| HNCD15 | *C. fructicola* | KJ132325 | KJ132126 | KJ131927 | KJ131728 |
| HNLY1 | *C. fructicola* | KJ132326 | KJ132127 | KJ131928 | KJ131729 |
| HNLY3 | *C. fructicola* | KJ132327 | KJ132128 | KJ131929 | KJ131730 |
| HNLY4 | *C. fructicola* | KJ132328 | KJ132129 | KJ131930 | KJ131731 |
| HNLY6 | *C. fructicola* | KJ132329 | KJ132130 | KJ131931 | KJ131732 |
| HNLY7 | *C. fructicola* | KJ132330 | KJ132131 | KJ131932 | KJ131733 |
| HNLY8 | *C. fructicola* | KJ132331 | KJ132132 | KJ131933 | KJ131734 |
| HNLY9 | *C. fructicola* | KJ132332 | KJ132133 | KJ131934 | KJ131735 |
| HNLY11 | *C. fructicola* | KJ132333 | KJ132134 | KJ131935 | KJ131736 |
| HNLY13 | *C. fructicola* | KJ132334 | KJ132135 | KJ131936 | KJ131737 |
| HNLY17 | *C. fructicola* | KJ132335 | KJ132136 | KJ131937 | KJ131738 |
| HNLY18 | *C. fructicola* | KJ132336 | KJ132137 | KJ131938 | KJ131739 |
| HNLY19 | *C. fructicola* | KJ132337 | KJ132138 | KJ131939 | KJ131740 |
| HNLY20 | *C. fructicola* | KJ132338 | KJ132139 | KJ131940 | KJ131741 |
| HNLY21 | *C. fructicola* | KJ132339 | KJ132140 | KJ131941 | KJ131742 |
| HNLY22 | *C. fructicola* | KJ132340 | KJ132141 | KJ131942 | KJ131743 |
| HNLY23 | *C. fructicola* | KJ132341 | KJ132142 | KJ131943 | KJ131744 |
| HNLY24 | *C. fructicola* | KJ132342 | KJ132143 | KJ131944 | KJ131745 |
| HNLY26 | *C. fructicola* | KJ132343 | KJ132144 | KJ131945 | KJ131746 |
| HNLY28 | *C. fructicola* | KJ132344 | KJ132145 | KJ131946 | KJ131747 |
| HNLY29 | *C. fructicola* | KJ132345 | KJ132146 | KJ131947 | KJ131748 |
| HNLY30 | *C. fructicola* | KJ132346 | KJ132147 | KJ131948 | KJ131749 |
| HNLY31 | *C. fructicola* | KJ132347 | KJ132148 | KJ131949 | KJ131750 |
| HNLY32 | *C. fructicola* | KJ132348 | KJ132149 | KJ131950 | KJ131751 |
| HNLY33 | *C. fructicola* | KJ132349 | KJ132150 | KJ131951 | KJ131752 |
| HNLY34 | *C. fructicola* | KJ132350 | KJ132151 | KJ131952 | KJ131753 |
| HNLY35 | *C. fructicola* | KJ132351 | KJ132152 | KJ131953 | KJ131754 |
| HNLY36 | *C. fructicola* | KJ132352 | KJ132153 | KJ131954 | KJ131755 |
| HNHH1 | *C. fructicola* | KJ132353 | KJ132154 | KJ131955 | KJ131756 |
| HNHH3 | *C. fructicola* | KJ132354 | KJ132155 | KJ131956 | KJ131757 |
| HNHH4 | *C. fructicola* | KJ132355 | KJ132156 | KJ131957 | KJ131758 |
| HNHH7 | *C. fructicola* | KJ132356 | KJ132157 | KJ131958 | KJ131759 |
| HNHH8 | *C. fructicola* | KJ132357 | KJ132158 | KJ131959 | KJ131760 |
| HNHH10 | *C. fructicola* | KJ132358 | KJ132159 | KJ131960 | KJ131761 |
| HNHH11 | *C. fructicola* | KJ132359 | KJ132160 | KJ131961 | KJ131762 |
| HNHH12 | *C. fructicola* | KJ132360 | KJ132161 | KJ131962 | KJ131763 |
| HNHH14 | *C. fructicola* | KJ132361 | KJ132162 | KJ131963 | KJ131764 |
| HNHH15 | *C. gloeosporides* | KJ132174 | KJ131975 | KJ131776 | KJ131577 |
| HNHH16 | *C. fructicola* | KJ132362 | KJ132163 | KJ131964 | KJ131765 |
| HNHH17 | *C. fructicola* | KJ132363 | KJ132164 | KJ131965 | KJ131766 |
| HNHH18 | *C. fructicola* | KJ132364 | KJ132165 | KJ131966 | KJ131767 |
| HNHH19 | *C. fructicola* | KJ132365 | KJ132166 | KJ131967 | KJ131768 |
| HNHH20 | *C. fructicola* | KJ132366 | KJ132167 | KJ131968 | KJ131769 |
| HNHH22 | *C. fructicola* | KJ132367 | KJ132168 | KJ131969 | KJ131770 |
| HNHH23 | *C. siamense* | KJ132197 | KJ131998 | KJ131799 | KJ131600 |
| HNHH24 | *C. siamense* | KJ132185 | KJ131986 | KJ131787 | KJ131588 |
| GXNN1 | [*Colletotrichum* sp.](http://www.ncbi.nlm.nih.gov/nuccore/KJ132176.1) | KJ132175 | KJ131976 | KJ131777 | KJ131578 |
| GXNN2 | *C. fructicola* | KJ132201 | KJ132002 | KJ131803 | KJ131604 |
| GXNN3 | *C. siamense* | KJ132192 | KJ131993 | KJ131794 | KJ131595 |
| GXNN4 | *C. fructicola* | KJ132202 | KJ132003 | KJ131804 | KJ131605 |
| GXNN5 | *C. siamense* | KJ132193 | KJ131994 | KJ131795 | KJ131596 |
| HNWZS2 | *C. fructicola* | KJ132203 | KJ132004 | KJ131805 | KJ131606 |
| HNWZS3 | *C. fructicola* | KJ132204 | KJ132005 | KJ131806 | KJ131607 |
| HNWZS4 | *C. fructicola* | KJ132205 | KJ132006 | KJ131807 | KJ131608 |
| HNWZS6 | *C. fructicola* | KJ132206 | KJ132007 | KJ131808 | KJ131609 |
| HNWZS7 | *C. fructicola* | KJ132207 | KJ132008 | KJ131809 | KJ131610 |
| FJLY1 | *C. fructicola* | KJ132208 | KJ132009 | KJ131810 | KJ131611 |
| FJLY2 | *C. fructicola* | KJ132209 | KJ132010 | KJ131811 | KJ131612 |
| FJLY5 | *C. fructicola* | KJ132210 | KJ132011 | KJ131812 | KJ131613 |
| FJLY7 | *C. fructicola* | KJ132211 | KJ132012 | KJ131813 | KJ131614 |
| FJLY8 | *C. fructicola* | KJ132212 | KJ132013 | KJ131814 | KJ131615 |
| FJLY9 | *C. fructicola* | KJ132213 | KJ132014 | KJ131815 | KJ131616 |
| FJLY10 | *C. fructicola* | KJ132214 | KJ132015 | KJ131816 | KJ131617 |
| FJLY11 | *C. fructicola* | KJ132215 | KJ132016 | KJ131817 | KJ131618 |
| FJLY15 | *C. fructicola* | KJ132216 | KJ132017 | KJ131818 | KJ131619 |
| FJLY16 | *C. fructicola* | KJ132217 | KJ132018 | KJ131819 | KJ131620 |
| FJLY20 | *C. fructicola* | KJ132218 | KJ132019 | KJ131820 | KJ131621 |
