# Supplementary material for: Population Genetic Analyses of the Fungal Pathogen Colletotrichum fructicola on Tea-Oil Trees in China
Source: PLoS One. 2016 Jun 14;11(6):e0156841. doi: 10.1371/journal.pone.0156841 (PMC4907445; doi:10.1371/journal.pone.0156841)
Supplement: S2 Table — Highlighted samples were excluded from population genetic analyses. For each isolate, the string of capital letters indicates its geographic location corresponding to those in Table 1. (DOCX) [file pone.0156841.s008.docx]

S2 Table. Allelic and genotype data for all 167 isolates of *C. fructicola* from southern China. Highlighted samples were excluded from population genetic analyses due to the small sample sizes from these populations. For each isolate, the string of capital letters indicates its geographic location corresponding to those in Table 1.

| Sample | ITS | CL | GS | GD | Genotype |
| --- | --- | --- | --- | --- | --- |
| CQXS1 | 5 | 3 | 6 | 8 | 19 |
| CQXS11 | 5 | 3 | 6 | 8 | 19 |
| CQXS16 | 1 | 3 | 11 | 5 | 1 |
| CQXS18 | 5 | 3 | 7 | 8 | 26 |
| CQXS3 | 5 | 3 | 6 | 5 | 16 |
| CQXS8 | 5 | 3 | 6 | 5 | 16 |
| CQXS9 | 5 | 3 | 6 | 8 | 19 |
| FJLY1 | 5 | 3 | 6 | 1 | 15 |
| FJLY10 | 5 | 3 | 5 | 5 | 11 |
| FJLY11 | 5 | 3 | 5 | 5 | 11 |
| FJLY15 | 5 | 3 | 5 | 6 | 12 |
| FJLY16 | 5 | 3 | 5 | 6 | 12 |
| FJLY2 | 5 | 3 | 5 | 6 | 12 |
| FJLY20 | 5 | 3 | 6 | 10 | 21 |
| FJLY5 | 5 | 3 | 5 | 6 | 12 |
| FJLY7 | 9 | 3 | 6 | 6 | 42 |
| FJLY8 | 5 | 3 | 5 | 6 | 12 |
| FJLY9 | 5 | 3 | 5 | 10 | 14 |
| GXNN2 | 5 | 3 | 5 | 6 | 12 |
| GXNN4 | 5 | 3 | 5 | 6 | 12 |
| HBSZ1 | 5 | 3 | 7 | 6 | 25 |
| HBSZ12 | 5 | 3 | 7 | 5 | 24 |
| HBSZ14 | 14 | 3 | 7 | 5 | 48 |
| HBSZ15 | 14 | 3 | 7 | 8 | 50 |
| HBSZ17 | 14 | 3 | 7 | 5 | 48 |
| HBSZ2 | 14 | 3 | 7 | 6 | 49 |
| HBSZ20 | 5 | 3 | 3 | 8 | 9 |
| HBSZ21 | 5 | 3 | 7 | 5 | 24 |
| HBSZ3 | 14 | 3 | 7 | 9 | 51 |
| HBSZ4 | 14 | 3 | 7 | 9 | 51 |
| HBSZ5 | 5 | 3 | 7 | 5 | 24 |
| HBSZ6 | 5 | 3 | 7 | 9 | 27 |
| HBSZ7 | 13 | 3 | 7 | 6 | 47 |
| HBSZ9 | 5 | 3 | 7 | 9 | 27 |
| HBXG1 | 5 | 3 | 10 | 9 | 33 |
| HBXG3 | 5 | 3 | 7 | 9 | 27 |
| HBXG4 | 5 | 3 | 7 | 10 | 28 |
| HBXG5 | 5 | 3 | 6 | 6 | 17 |
| HNCD11 | 10 | 3 | 7 | 9 | 43 |
| HNCD15 | 5 | 5 | 6 | 5 | 36 |
| HNCD8 | 15 | 3 | 6 | 9 | 53 |
| HNCD9 | 5 | 5 | 6 | 6 | 37 |
| HNHH1 | 5 | 3 | 7 | 6 | 25 |
| HNHH10 | 5 | 3 | 6 | 8 | 19 |
| HNHH11 | 5 | 3 | 5 | 8 | 13 |
| HNHH12 | 5 | 3 | 6 | 5 | 16 |
| HNHH14 | 5 | 3 | 6 | 9 | 20 |
| HNHH16 | 5 | 3 | 6 | 9 | 20 |
| HNHH17 | 5 | 3 | 6 | 11 | 22 |
| HNHH18 | 5 | 3 | 6 | 5 | 16 |
| HNHH19 | 5 | 3 | 7 | 8 | 26 |
| HNHH20 | 5 | 3 | 6 | 9 | 20 |
| HNHH22 | 5 | 3 | 5 | 6 | 12 |
| HNHH3 | 12 | 3 | 6 | 9 | 46 |
| HNHH4 | 5 | 3 | 7 | 6 | 25 |
| HNHH7 | 5 | 3 | 7 | 10 | 28 |
| HNHH8 | 5 | 3 | 5 | 10 | 14 |
| HNLY1 | 5 | 3 | 6 | 9 | 20 |
| HNLY11 | 3 | 3 | 6 | 9 | 3 |
| HNLY13 | 6 | 3 | 6 | 9 | 39 |
| HNLY17 | 5 | 3 | 6 | 6 | 17 |
| HNLY18 | 5 | 3 | 7 | 8 | 26 |
| HNLY19 | 5 | 3 | 6 | 8 | 19 |
| HNLY20 | 5 | 3 | 7 | 8 | 26 |
| HNLY21 | 5 | 3 | 6 | 8 | 19 |
| HNLY22 | 5 | 3 | 6 | 8 | 19 |
| HNLY23 | 5 | 3 | 7 | 8 | 26 |
| HNLY24 | 5 | 3 | 7 | 4 | 23 |
| HNLY26 | 5 | 3 | 6 | 8 | 19 |
| HNLY28 | 5 | 3 | 7 | 5 | 24 |
| HNLY29 | 5 | 3 | 7 | 10 | 28 |
| HNLY3 | 5 | 3 | 6 | 6 | 17 |
| HNLY30 | 5 | 3 | 7 | 5 | 24 |
| HNLY31 | 5 | 3 | 7 | 5 | 24 |
| HNLY32 | 5 | 3 | 7 | 8 | 26 |
| HNLY33 | 5 | 3 | 8 | 2 | 29 |
| HNLY34 | 5 | 3 | 6 | 8 | 19 |
| HNLY35 | 5 | 3 | 8 | 8 | 30 |
| HNLY36 | 5 | 3 | 6 | 8 | 19 |
| HNLY4 | 5 | 3 | 6 | 9 | 20 |
| HNLY6 | 5 | 3 | 6 | 9 | 20 |
| HNLY7 | 5 | 3 | 6 | 9 | 20 |
| HNLY8 | 5 | 3 | 6 | 9 | 20 |
| HNLY9 | 5 | 3 | 8 | 9 | 31 |
| HNMJH1 | 5 | 3 | 7 | 9 | 27 |
| HNMJH12 | 6 | 3 | 6 | 6 | 38 |
| HNMJH13 | 5 | 3 | 6 | 9 | 20 |
| HNMJH17 | 5 | 3 | 6 | 8 | 19 |
| HNMJH18 | 5 | 3 | 6 | 8 | 19 |
| HNMJH2 | 5 | 4 | 6 | 6 | 35 |
| HNMJH20 | 11 | 4 | 6 | 8 | 45 |
| HNMJH23 | 5 | 3 | 7 | 10 | 28 |
| HNMJH3 | 5 | 3 | 7 | 9 | 27 |
| HNMJH7 | 5 | 3 | 7 | 9 | 27 |
| HNMJH8 | 5 | 3 | 6 | 9 | 20 |
| HNTJL1 | 5 | 3 | 9 | 9 | 32 |
| HNTJL13 | 5 | 3 | 6 | 9 | 20 |
| HNTJL14 | 5 | 3 | 2 | 3 | 8 |
| HNTJL16 | 5 | 3 | 6 | 9 | 20 |
| HNTJL17 | 2 | 3 | 6 | 5 | 2 |
| HNTJL2 | 4 | 3 | 7 | 9 | 4 |
| HNTJL21 | 5 | 3 | 6 | 5 | 16 |
| HNTJL3 | 5 | 3 | 10 | 9 | 33 |
| HNTJL4 | 5 | 3 | 6 | 5 | 16 |
| HNTJL6 | 5 | 3 | 6 | 6 | 17 |
| HNTJL7 | 5 | 3 | 6 | 9 | 20 |
| HNTJL9 | 15 | 3 | 6 | 6 | 52 |
| HNWZS2 | 5 | 3 | 1 | 1 | 7 |
| HNWZS3 | 5 | 3 | 12 | 1 | 34 |
| HNWZS4 | 5 | 3 | 1 | 1 | 7 |
| HNWZS6 | 5 | 3 | 6 | 1 | 15 |
| HNWZS7 | 7 | 3 | 12 | 1 | 40 |
| HNYX1 | 5 | 3 | 7 | 6 | 25 |
| HNYX10 | 5 | 3 | 6 | 8 | 19 |
| HNYX11 | 5 | 3 | 6 | 8 | 19 |
| HNYX2 | 5 | 3 | 7 | 9 | 27 |
| HNYX4 | 5 | 3 | 6 | 6 | 17 |
| HNYX7 | 8 | 3 | 6 | 6 | 41 |
| JXCB1 | 5 | 3 | 6 | 6 | 17 |
| JXCB11 | 5 | 3 | 7 | 8 | 26 |
| JXCB12 | 5 | 3 | 5 | 5 | 11 |
| JXCB13 | 5 | 3 | 7 | 8 | 26 |
| JXCB15 | 5 | 3 | 5 | 5 | 11 |
| JXCB16 | 5 | 3 | 6 | 7 | 18 |
| JXCB18 | 5 | 3 | 7 | 5 | 24 |
| JXCB19 | 5 | 3 | 7 | 5 | 24 |
| JXCB20 | 5 | 3 | 5 | 5 | 11 |
| JXCB22 | 5 | 3 | 5 | 5 | 11 |
| JXCB23 | 5 | 3 | 5 | 5 | 11 |
| JXCB24 | 5 | 3 | 6 | 5 | 16 |
| JXCB25 | 5 | 3 | 6 | 8 | 19 |
| JXCB26 | 5 | 3 | 7 | 8 | 26 |
| JXCB27 | 5 | 1 | 6 | 5 | 5 |
| JXCB29 | 5 | 3 | 6 | 5 | 16 |
| JXCB3 | 5 | 3 | 7 | 9 | 27 |
| JXCB31 | 5 | 3 | 5 | 10 | 14 |
| JXCB32 | 5 | 3 | 6 | 8 | 19 |
| JXCB4 | 5 | 3 | 5 | 6 | 12 |
| JXCB5 | 5 | 3 | 6 | 6 | 17 |
| JXCB6 | 5 | 3 | 6 | 11 | 22 |
| JXCB8 | 5 | 3 | 5 | 10 | 14 |
| JXCB9 | 5 | 3 | 7 | 5 | 24 |
| JXGS-A1 | 5 | 3 | 6 | 9 | 20 |
| JXGS-A10 | 5 | 3 | 6 | 5 | 16 |
| JXGS-A11 | 5 | 3 | 6 | 8 | 19 |
| JXGS-A12 | 5 | 3 | 5 | 8 | 13 |
| JXGS-A14 | 5 | 3 | 6 | 10 | 21 |
| JXGS-A15 | 5 | 3 | 5 | 5 | 11 |
| JXGS-A17 | 5 | 3 | 6 | 5 | 16 |
| JXGS-A18 | 5 | 3 | 4 | 5 | 10 |
| JXGS-A2 | 5 | 3 | 7 | 9 | 27 |
| JXGS-A20 | 5 | 3 | 7 | 8 | 26 |
| JXGS-A21 | 11 | 3 | 6 | 8 | 44 |
| JXGS-A22 | 5 | 3 | 6 | 8 | 19 |
| JXGS-A23 | 5 | 3 | 5 | 5 | 11 |
| JXGS-A24 | 5 | 3 | 6 | 5 | 16 |
| JXGS-A25 | 5 | 3 | 8 | 8 | 30 |
| JXGS-A29 | 5 | 3 | 6 | 8 | 19 |
| JXGS-A3 | 5 | 3 | 7 | 10 | 28 |
| JXGS-A4 | 5 | 3 | 5 | 5 | 11 |
| JXGS-A5 | 5 | 3 | 7 | 5 | 24 |
| JXGS-B13 | 5 | 2 | 6 | 5 | 6 |
| JXGS-B14 | 5 | 3 | 6 | 8 | 19 |
| JXGS-B3 | 5 | 3 | 5 | 5 | 11 |
| JXGS-B5 | 5 | 3 | 6 | 5 | 16 |
| JXGS-B6 | 5 | 3 | 6 | 5 | 16 |
| JXGS-B7 | 5 | 3 | 6 | 8 | 19 |
