# Supplementary material for: Population Genetic Analyses of the Fungal Pathogen Colletotrichum fructicola on Tea-Oil Trees in China
Source: PLoS One. 2016 Jun 14;11(6):e0156841. doi: 10.1371/journal.pone.0156841 (PMC4907445; doi:10.1371/journal.pone.0156841)
Supplement: S3 Table — (DOCX) [file pone.0156841.s009.docx]

S3 Table. Output from DIYABC analyses showing the relative likelihood of the two scenarios of population history of *C. fructicola* in southern China. Both the direct approach and the logistic approach favored Scenario #1 over Scenario #2.

Direct Approach

Closest Scenario 1 Scenario 2

100 0.6100 [0.3077,0.9123] 0.3900 [0.0877,0.6923]

200 0.5300 [0.2207,0.8393] 0.4700 [0.1607,0.7793]

300 0.5167 [0.2069,0.8264] 0.4833 [0.1736,0.7931]

400 0.5000 [0.1901,0.8099] 0.5000 [0.1901,0.8099]

500 0.5080 [0.1981,0.8179] 0.4920 [0.1821,0.8019]

600 0.5183 [0.2086,0.8280] 0.4817 [0.1720,0.7914]

700 0.5357 [0.2266,0.8448] 0.4643 [0.1552,0.7734]

800 0.5700 [0.2631,0.8769] 0.4300 [0.1231,0.7369]

900 0.5889 [0.2839,0.8939] 0.4111 [0.1061,0.7161]

1000 0.5860 [0.2807,0.8913] 0.4140 [0.1087,0.7193]

Logistic approach

N Scenario 1 Scenario 2

100 1.0000 [1.0000,1.0000] 0.0000 [0.0000,0.0000]

200 1.0000 [1.0000,1.0000] 0.0000 [0.0000,0.0000]

300 1.0000 [1.0000,1.0000] 0.0000 [0.0000,0.0000]

400 1.0000 [1.0000,1.0000] 0.0000 [0.0000,0.0000]

500 1.0000 [1.0000,1.0000] 0.0000 [0.0000,0.0000]

600 1.0000 [1.0000,1.0000] 0.0000 [0.0000,0.0000]

700 1.0000 [1.0000,1.0000] 0.0000 [0.0000,0.0000]

800 1.0000 [1.0000,1.0000] 0.0000 [0.0000,0.0000]

900 1.0000 [1.0000,1.0000] 0.0000 [0.0000,0.0000]

1000 1.0000 [1.0000,1.0000] 0.0000 [0.0000,0.0000]
